# Heating dictates the scalability of CO_2_ electrolyzer types†

**DOI:** 10.1039/d4ey00190g

**Published:** 2024-12-27

**Authors:** Jan-Willem Hurkmans, Henri M. Pelzer, Tom Burdyny, Jurriaan Peeters, David A. Vermaas

**Affiliations:** a Department of Chemical Engineering, Delft University of Technology 2629 HZ Delft The Netherlands D.A.Vermaas@tudelft.nl; b Process & Energy Department, Delft University of Technology 2628 CB Delft The Netherlands

## Abstract

Electrochemical CO_2_ reduction offers a promising method of converting renewable electrical energy into valuable hydrocarbon compounds vital to hard-to-abate sectors. Significant progress has been made on the lab scale, but scale-up demonstrations remain limited. Because of the low energy efficiency of CO_2_ reduction, we suspect that significant thermal gradients may develop in industrially relevant dimensions. We describe here a model prediction for non-isothermal behavior beyond the typical 1D models to illustrate the severity of heating at larger scales. We develop a 2D model for two membrane electrode assembly (MEA) CO_2_ electrolyzers; a liquid anolyte fed MEA (exchange MEA) and a fully gas fed configuration (full MEA). Our results indicate that full MEA configurations exhibit very poor electrochemical performance at moderately larger scales due to non-isothermal effects. Heating results in severe membrane dehydration, which induces large Ohmic losses in the membrane, resulting in a sharp decline in the current density along the flow direction. In contrast, the anolyte employed in the exchange MEA configuration is effective in preventing large thermal gradients. Membrane dehydration is not a problem for the exchange MEA configuration, leading to a nearly constant current density over the entire length of the modeled domain, and indicating that exchange MEA configurations are well suited for scale-up. Our results additionally indicate that a balance between faster kinetics, higher ionic conductivity, smaller pH gradients and lower CO_2_ solubility causes an optimum operating temperature between 60 and 70 °C.

## Abstract 2

Electrochemical CO_2_ reduction offers a promising method of converting renewable electrical energy into valuable hydrocarbon compounds, but upscaling generates substantial heat that poses limitations to the cell design.

## Graphical abstract

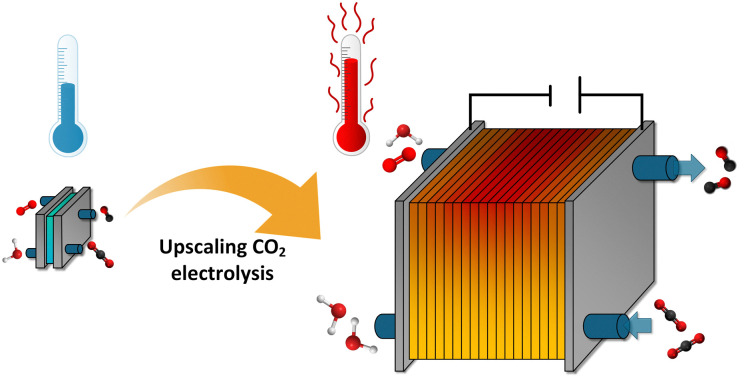

**Broader context**

In order to meet the goals set by the European Union to become climate-neutral by 2050, the industrial- and transport sectors will have to be de-fossilized. Electrification and implementation of hydrogen shows great potential in replacing fossil fuels, however certain hard-to-abate sectors such as airline travel and chemical manufacturing are very challenging to decarbonize and projected to still require hydrocarbon sources in 2050. These necessary hydrocarbon chemicals and fuels, responsible for 10–20% of the global greenhouse gas emission, can be produced sustainably by the electrolysis of captured CO_2_ using renewable electricity. While the figures for renewable hydrocarbon fuels (e-liquids) and carbon-based chemicals (methanol, ethylene, *etc.*) are astronomical and urgent, the knowledge about CO_2_ electrolysis upscaling is very limited. Heat management will be a crucial factor for successfully upscaling CO_2_ electrolysis, as the high overpotentials for CO_2_ reduction render approximately 50% of the electrolyzer's energy input to heat, and the reaction kinetics and CO_2_ solubility are sensitive to temperature. Therefore, understanding heat sources and temperature distributions are a necessity for developing the urgently demanded electrochemical CO_2_ conversion.

## Introduction

Hydrocarbon chemicals and fuels can be produced sustainably by the electrolysis of captured CO_2_ using renewable electricity.^1,2^ CO_2_ electrolyzers, to synthesize a variety of products including CO, formate or C_2+_ hydrocarbons, have seen significant developments over the last two decades and reports of industrially relevant current densities become more and more frequent on the lab scale.^3–6^ As the geometric size of CO_2_ electrolyzers increases, however, additional factors must be considered to enable lab-scale performance demonstrations to be replicated for larger systems. Many previous works have already identified and addressed operational issues such as mass transport limitations at high current density,^7–9^ flooding and pressure control for taller electrolyzers,^10,11^ salt precipitation and CO_2_ crossover.^12–16^ One aspect not well-considered so far is how the substantial amount of heat generated in a CO_2_ electrolyzer will impact operation at larger scales. To determine these effects the heat- and temperature distributions inside a CO_2_ electrolyzer are required, which are currently largely unknown.

We expect that the generation of heat in large-scale CO_2_ electrolyzers can cause more substantial constraints compared to *e.g.* fuel cells and PEM electrolyzers, as CO_2_ electroreduction suffers from high overpotentials and homogeneous reactions in the anolyte- and ionomer-phase add an additional source of heat. Moreover, large interfacial- and Ohmic losses can be expected within these electrochemical cells since components such as membranes have not been tailored for CO_2_ reduction.^17^ Combined with the inherent poor heat transfer characteristics of gas diffusion electrode (GDE) configurations, it is important to understand where large thermal gradients exist and if these limit system performance or scalability. Accordingly, it is important to understand and predict heating phenomena in order to realize CO_2_ electrolysis on an industrial scale.

It is understood that many aspects of the electroreduction of CO_2_ are influenced by temperature as shown by experimental studies using different initial operating conditions. Löwe *et al.* varied the operating temperature for a GDE based system between 20 and 70 °C finding an optimum performance at 50 °C.^18^ The authors attribute this optimum to the fact that while mass transport and the kinetics are enhanced at higher temperatures, the solubility of CO_2_ in water significantly decreases with temperature. Similar optima were observed for the electroreduction of CO_2_ using membrane electrode assembly (MEA) configurations employing poly(aryl piperidinium)-based anion exchange membranes (AEM) and alkaline polymer electrolyte membranes with pure water as electrolyte.^3,19^ The magnitude of heating at industrially relevant conditions has only been reported in two modeling studies by Weng *et al.*^20,21^ However, these models are implemented in a one-dimensional domain which cannot fully capture the extent of the non-isothermal effects due to the exclusion of down-the-channel effects which have been shown to vary significantly.^22,23^ Thus the temperature development along a flow channel which can impact hydration, current density distribution and CO_2_ solubility have yet to be understood.

In this study, we develop a two-dimensional model displaying large-scale heating effects of an electrolyzer, with an emphasis on the temperature profile from inlet to outlet. We aim to compare temperature distributions for two AEM-based MEA configurations (full MEA and exchange MEA), producing CO from CO_2_, utilizing an IrO_2_ anode and an Ag cathode, respectively, to investigate their thermal scalability. In our model, CO is considered the only product for CO_2_R competing with the hydrogen evolution reaction (HER). However, the thermoneutral potential of CO_2_R to CO is in the same order of magnitude as the thermoneutral potential of other gaseous CO_2_R products like ethylene (C_2_H_4_) implying similar heating rates for MEA configurations aiming to produce other gaseous products than CO.^24^

The model underlines the significance of heating and thus the analysis of non-isothermal effects, necessary to further understand the multi-scale phenomena at play in CO_2_ electrolyzers. Our results demonstrate that the temperature within the cell can differ more than 10 °C in a 20 cm tall cell, depending on the MEA configuration and current density.

## Model development

**Fig. 1:**
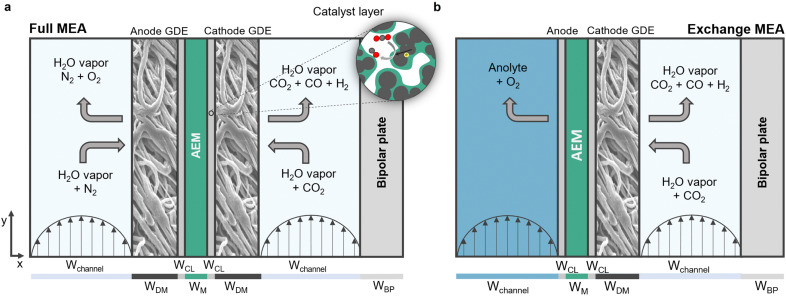
Schematic representations of the computational domains used in this work. The feed flows in the *y*-direction whereby the serpentine motion is neglected for simplicity. (a) Full MEA configuration is fed with a gaseous feed at both anode and cathode. (b) Exchange MEA configuration is fed liquid electrolyte at the anode side. The cathode compartment is fed humidified CO_2_. Details regarding dimensions are provided in the ESI,† Table S8.

The Multiphysics model developed in this work is an expansion based on the seminal one dimensional model developed by Weng *et al.* for CO_2_ MEA electrolyzers.^20,21^ We consider two 2D electrolyzer configurations schematically shown below; an exchange MEA which is supplied with saturated 0.5 M KHCO_3_ electrolyte to the anode and a full MEA which is supplied with fully humidified gas (Fig. 1a and b). This choice of electrolyte and electrode configuration is to illustrate the cases with minimal resistance, which can be considered as conservative heating scenarios.

In order to simulate an MEA configuration in a scaled-up setting, the computational domain includes bipolar plates that separate MEA cells. Periodic boundary conditions for the temperature field are implemented for the outer boundaries along the *y*-direction. This resembles a stack that is sufficiently large to neglect heat dissipation *via* the walls. The flow path of the model is 20 cm, whereby the serpentine geometry is neglected which means that the depth of the flow channel is infinite. We recognize that not taking into account the three-dimensional geometry will result in discrepancies with heat transfer in reality, our goal however is to give an idea of the heat development in an idealized electrolyzer. Non-steady-state phenomena of importance like flooding and salt formation are out of the scope of this work and thus not covered. Further, the model neglects contact resistances as the used configuration most closely resembles a catalyst-coated membrane (CCM). The model validation as well as an in depth comparison between the model and experimental work can be seen in Section 1 of the ESI.†

The gaseous compartments contain GDEs constructed of a fibrous diffusion media through which the gaseous species diffuse to- and from the catalytic layers. The catalytic layers are a very thin multiphase domain which consists of a mixture of anion exchange ionomer, catalytic particles and void whereby it is assumed that the catalytic particles are homogeneously coated with a thin film of the ionomer which acts as the electrolyte as it contains a significant amount of water. At the surface of the catalytic particles, heterogeneous electrochemical reactions take place (Fig. 1a).

The porous domains are modeled as a volume averaged medium, thereby ignoring local heterogeneities. While exchange MEA configurations historically use a mesh as the anode, the anode in this work resembles a catalyst coated membrane which has shown to greatly improve electrochemical performance.^3,4^ The two catalytic layers sandwich an AEM which allows for an ionic current due to the simultaneous diffusion and migration of dissolved charged species. We apply a volumetric flow rate of CO_2_ of 50 SCCM per cell, which is sufficient to reach high faradaic efficiencies considering the dimensions of our models and the maximum investigated current density.^25^ For a serpentine flow channel, this translates to an average gas velocity of approximately 3 m s^−1^ for a cross-sectional area of 0.25 mm^2^. The liquid flowrate is taken as 5 SCCM. The following sections will present the key assumptions and governing equations (variables and governing equations that are not essential with respect to the results are provided in Section 7 of the ESI†). General parameters regarding operating conditions are provided in Table 1. Details regarding dimensions, parameters and variables are provided in Table S8 (ESI†). A comparison to the model by Weng *et al.* and to experimental work is given in Section 1 of the ESI.† Details regarding the meshes used in the simulations are provided in ESI,† Section 5.

**Table 1:** Operational parameters used in the simulations for the base case

Parameter	Description	Value	Unit	Ref.
*P*_op_	Operating pressure	1	atm	—
*T*_op_	Operating (inlet) temperature	20	°C	—
*ū*_l_	Average liquid velocity	0.3	m s^−1^	—
*ū*_g_	Average gas velocity	3	m s^−1^	—
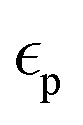	Porosity CL	0.675	—	26
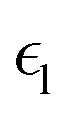	Ionomer fraction CL	0.225	—	[ESI, Section 7.5]
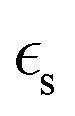	Solid fraction CL	0.1	—	[ESI, Section 7.5]
*a*_v,CL_	Active specific surface area CL	6 × 10^6^	m^−1^	26
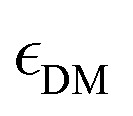	Porosity diffusion medium	0.8	—	27
*c*_KHCO_3__	Concentration anolyte	0.5	M	—

In order to simulate large scale electrolyzer behavior, an expansion method is employed which essentially decomposes the computational domain in small sub cells which are solved sequentially.^22^ The applied potential is kept constant over the entire geometry, which allows the current density to change along the channel length, capturing local variations across the electrode. The domains are electrically homogeneous, and we assume no contact resistance between them. A detailed description and validation of this approach is given in ESI,† Section 2.

In the anode catalytic domain we consider the oxygen evolution reaction (OER) on an IrO_2_ catalyst through both the acidic pathway and the alkaline pathway, respectively:

1 2H_2_O → O_2_ + 4H^+^ + 4e^−^

2 4OH^−^ → O_2_ + 2H_2_O + 4e^−^

For a silver catalyst, the carbon monoxide evolution reaction (COER) will be the prevalent charge transfer reaction in the cathode catalyst layer:^28^

3 CO_2_ + H_2_O + 2e^−^ → CO + 2OH^−^

In the cathode catalytic domain only the alkaline pathway of the parasitic hydrogen evolution (HER) is considered since the microenvironment in the catalytic layer is expected to have alkaline conditions:^29^

4 2H_2_O + 2e^−^ → H_2_ + 2OH^−^

The local current densities of the charge transfer reactions are computed through the concentration dependent Butler–Volmer equation. The local current density ***i*** [mA cm^−2^], for a charge transfer reaction *k*, reads:5
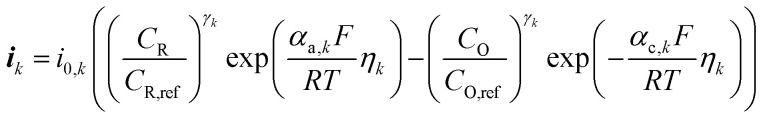
Wherein *C*_R_ and *C*_O_ [M] correspond to the concentration of the species undergoing reduction or oxidation, respectively. They are normalized by a reference concentration of 1 [M]. *α*_a,*k*_ and α_c,*k*_ [−] are the charge transfer coefficients for the anodic- and cathodic current respectively. *γ*_*k*_ [−] is the reaction order. *η*_*k*_ [V] is the overpotential for reaction *k* expressed as:

6 *η*_*k*_ = (*ϕ*_s_ − *ϕ*_l_) − *U*_eq,*k*_

Here, *ϕ*_l_ and *ϕ*_s_ [V] are the potentials in the electrolyte phase and the solid phase, respectively. *U*_eq,*k*_ [V] is the equilibrium potential of reaction *k* which is a function of the local pH in the microenvironment of the catalytic layer and can be approximated as:7
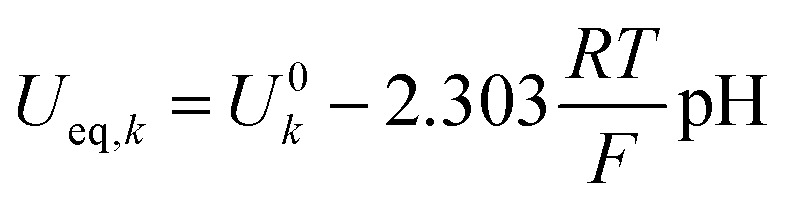
where *U*^0^_*k*_ [V] is the standard reduction potential for reaction *k.* We acknowledge the mistake identified by Nesbitt *et al.* for the use of the incorrect standard reduction potential of the COER in alkaline environments.^30^ However, since the kinetic parameters obtained from experimental results in this work have been deduced with this technically wrong value, the resulting kinetic parameters are consistent with experimental work. *i*_0,*k*_ [mA cm^−2^] represents the exchange current density for reaction *k* which follows an Arrhenius type of dependency on temperature.^31–33^8
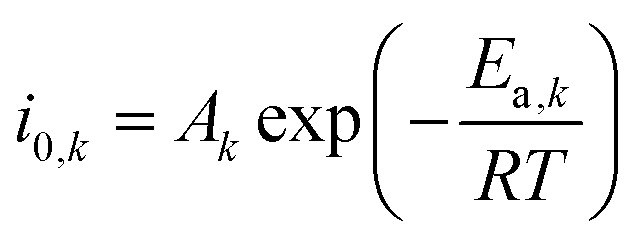
*A*_*k*_ [mA cm^−2^] is a fitted pre-exponent factor and *E*_a,*k*_ [kJ mol^−1^] is the apparent activation energy, which is dependent on the pH for the HER and the OER.^34–37^ The kinetic parameters in this study have been adopted from Weng *et al.* and are summarized in Table S8 (ESI†).^20^

The electric potential and the electrolyte potential required to solve for the current densities are determined by solving the following charge conservation equations for the electrolyte phase and the solid phase:

9 ***i***_l_ = −*σ*_l,eff_∇*ϕ*_l_ ***i***_s_ = −*σ*_s,eff_∇*ϕ*_s_

Here, ***i***_l_ and ***i***_s_ [mA cm^−2^] are the ionic and solid phase currents and *σ*_l,eff_ and *σ*_l,eff_ [S m^−1^] are the conductivities of the respective phases adjusted for porosity through a Bruggeman's correction. The ionic conductivity varies in space and follows from the local ion composition:10
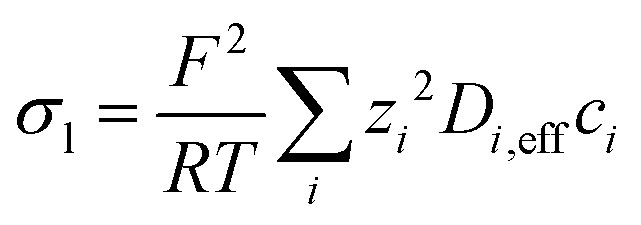
where *z*_*i*_ is the valence of a species *i* and *D*_*i*,eff_ the respective effective diffusion coefficient.

### Water transport in ionomer domains

The membrane is one of the key components within a MEA-based electrolyzer as it transports water, ions and thermal energy and its associated transport properties can vary greatly depending on operating conditions. As such, it can have major implications on the modeling results. We will follow the mathematical transport model for proton exchange membrane (PEM) transport by Weber *et al.*^38,39^ While CO_2_ electrolyzers typically employ AEMs, similar transport behavior has been observed experimentally.^40–42^ The transport model builds on the notion that two types of transport modes co-exist depending on whether the membrane is equilibrated with liquid water or with water vapor. The balance of these transport modes are dependent on the dimensionless water content of the ionomer, commonly denoted with *λ*. *λ* depends on the water activity, *a*_w_ [−], and the state of the membrane, which is expressed through the fraction of expanded channels, *S* [−]. The method used to compute *S*, and subsequently *λ*, is given in the ESI† (Section 7.3). The water activity *a*_w_, required for *λ*, is derived from the gradient in chemical potential of water in the ionomer phase, ∇*μ*_w_, defined as:11
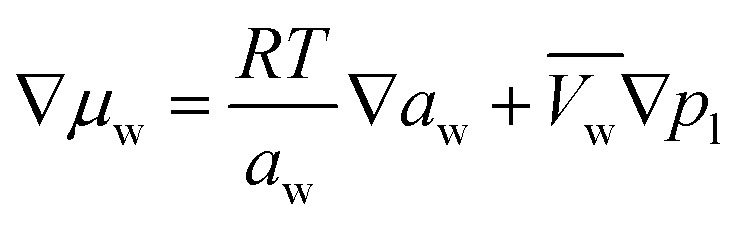
Wherein 
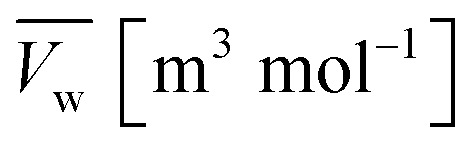
 is the molar volume of liquid water and *p*_l_ [Pa] is the liquid pressure relative to a reference pressure. The ∇*μ*_w_ is solved from the conservation equation for the water content:12
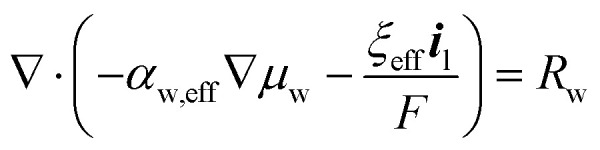
Here *ξ*_eff_ [−] is the overall effective electro-osmotic coefficient, *α*_w,eff_ [mol^2^ J^−1^ cm^−1^ s^−1^] is the effective water transport coefficient which is a function of the water content itself and has been linearly interpolated from Peng *et al.* with respect to temperature for vapor equilibrated membranes.^42^

### Transport and interactions of dissolved species

In the electrolyte and ionomer phase domains the concentrations of the species OH^−^, H^+^, HCO_3_^−^, CO_3_^2−^, K^+^ and CO_2_ [M] are simulated. The gaseous products found in eqn (1)–(4) have a very low solubility in water and are therefore neglected. The conservation of species *i* is computed with the steady state Nernst–Planck equation:13
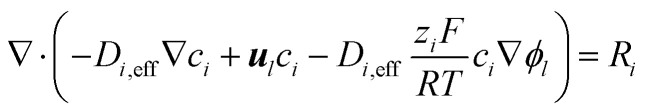
In the exchange MEA model, the electroneutrality constraint, 
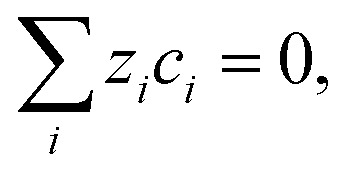
 is used to compute the concentration distribution of the inert ion K^+^ from the remaining species. For the full MEA model, this approach is problematic since the amount of K^+^ in a vapor-equilibrated AEM is finite and relatively small, resulting in negative K^+^ concentrations. In order to prevent charge separation and ensure physical concentrations everywhere, the Nernst–Planck equation is adjusted with an artificial charge separation limiting term to ensure a homogeneous charge distribution. Details and argumentation for this adjustment can be found in the ESI,† Section 3.

At the interface of electrolyte and the ionomer phase, a Donnan potential (Δ*ϕ*_*l*,Donnan_ [V]) arises which creates an energy barrier for oppositely charged species (relative to the membrane background charge) to enter, resulting in the partial exclusion of co-ions. The relationship used to relate the potential jump to the concentration difference of species *i* between electrolyte and ionomer phase is:14
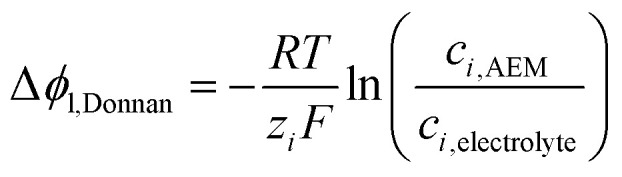
Since the full MEA configuration is not in contact with electrolyte, no Donnan potentials appear in this configuration. However, the initial conditions for the ionic species within the membrane must be determined through this relation by simulating membrane equilibration between the AEM and an electrolyte with a certain concentration. Numerical values for these initial values are provided in Table S8 (ESI†).

The temperature dependent diffusion coefficients, *D*_*i*,w_ [m^2^ s^−1^], in the liquid domain are given in Table S6 (ESI†). Inside the ionomer phase, the coefficients are corrected for the membrane water content, *λ*, following Grew *et al.*:^43,44^15
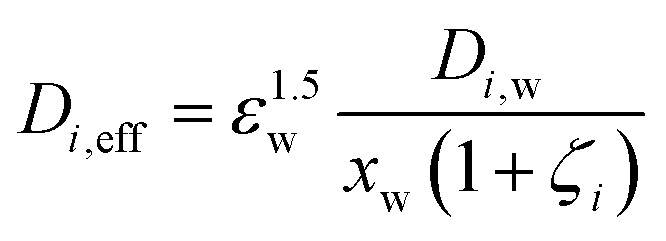
whereby *ε*_w_ and *x*_w_ are the water volume- and water molar fractions in the ionomer phase, respectively*. ζ*_*i*_ [−] is a parameter which describes the interaction of the dissolved species with either the membrane or with liquid water and has its origin in kinetic theory. The values of the parameters are dependent on *λ* and detailed expressions can be found in the ESI,† Section 7.8.

In the ionomer- and liquid domains, the following homogeneous reactions take place:16
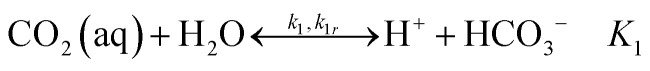
17
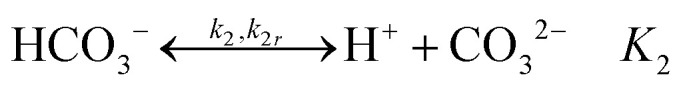
18
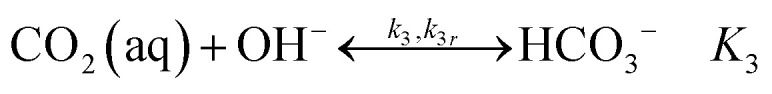
19
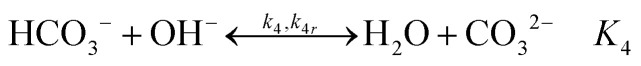
20
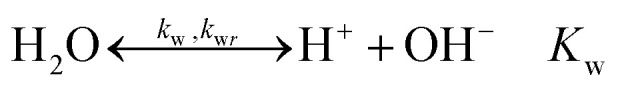
Expressions for the temperature dependent equilibrium constants *K*_1_, *K*_2_ and *K*_w_ are computed with empirically determined relations.^45,46^ Subsequently, the forward and backward rate constants are determined extrapolations of empirical relations from Schultz *et al.* (Table S7, ESI†).^47^

### Momentum and gaseous species transport

A Poiseuille flow profile is assumed for the velocity fields in the gas and liquid compartment (*u*_*y*_ = −6*ū*[(*x*/*W*_channel_)^2^ − *x*/*W*_channel_]), where *ū* [m s^−1^] is the average velocity. It is important to note that the evolution of gas bubbles in the liquid compartment will likely distort the parabolic velocity profile. Additionally, the presence of bubbles in this domain will affect the effective liquid properties. Nevertheless, the thermal properties in the exchange MEA cell configuration are mainly governed by the flow rate of the liquid electrolyte phase, which remains constant along the channel. Thus, bubble formation is expected to not strongly affect the average temperature profile and is therefore neglected, as the accurate simulation of bubble formation would extend the computational complexity of this work.

In the gaseous porous domains, the flow is computed with Darcy's law. The transport of gaseous species are solved with the Maxwell–Stefan model whereby the temperature dependence of the gaseous diffusion coefficients is incorporated using the framework of Fuller *et al.*^48^ For details regarding the governing equations concerning mass and momentum transport in the gas phase, the reader is pointed towards the ESI,† Section 7.1.

### Heat transfer

The steady state thermal energy balance summed over all domains can be written as:21
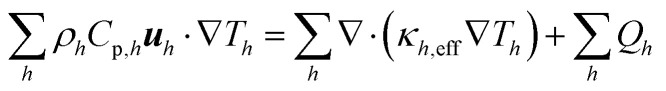
Wherein for a domain *h*, *ρ*_*h*_ [kg m^−3^] is the density, *C*_p,*h*_ [J kg^−1^ K^−1^] is the specific heat capacity and *u*_h_ is the velocity field. *κ*_*h*,eff_ [W m^−1^ K^−1^] is the effective conductivity within the domain which may consist of multiple phases. *Q*_h_ [W m^−3^] relates to the total heat source in domain *h* which is constituted by the heat sources listed below. The term on the left-hand side of the equation corresponds to advection, the first term on the right-hand side refers to conduction and the second term corresponds to heating and cooling. Five heat generation mechanisms are considered which vary per domain:

1. *Irreversible reaction heating* – The heat associated with the irreversible losses due to the activation potential of the charge transfer reaction *k* is:22
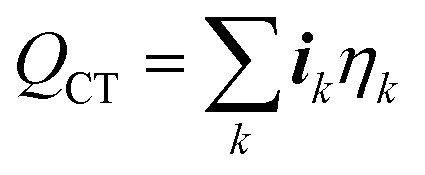


2. *Reaction entropy heating –* The heat related to the reversible entropy change of the charge transfer reaction *k* is:23
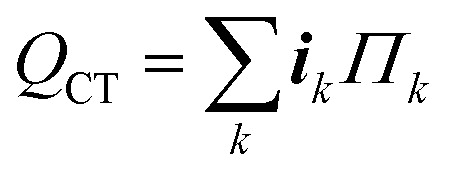
Here, *Π*_*k*_ [mV] is the Peltier coefficient, a measurable quantity which approximates the reversible heat generation associated with entropy changes of a half-cell reaction 
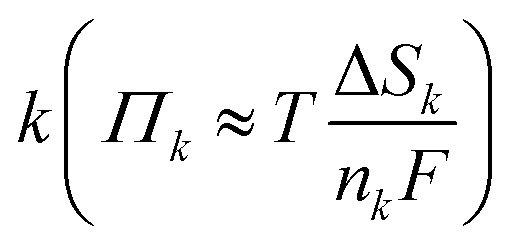
. The Peltier coefficients used in this modeling study are based on the values used by Weng *et al.*, who averaged the values of several experimental studies.^20,49^

3. *Ohmic heating* – The heat associated with resistive losses of the conducting medium are expressed as:24
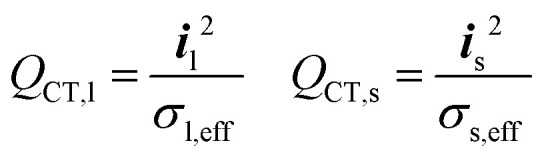
For the electrolytic- and solid phase, respectively.

4. *Enthalpy change of homogeneous reactions* – The heat associated with the homogeneous reactions (such as carbonate buffering) in the electrolyte domains are determined by the enthalpy change of said reaction determined by the Van't Hoff Equation for a homogeneous reaction *j*:25
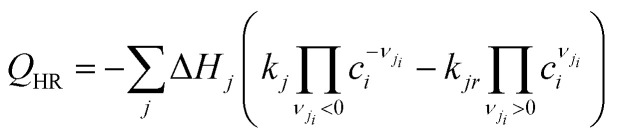
Whereby Δ*H*_*j*_ [kJ mol^−1^] is the enthalpy change of reaction *j* and *v*_*ji*_ [−] the stoichiometric coefficient of species *i* for reaction *j*.

5. *Heat of vaporization* – The enthalpy change related to the phase changes of water is accounted for with:

26 *Q*_PT_ = Δ*H*_w,vap_*R*_w,PT_

where Δ*H*_w,vap_ [kJ mol^−1^] is the temperature dependent enthalpy change of water vaporization and *R*_w,PT_ [mol m^−3^ s^−1^] is the vaporization rate of water (ESI,† Section 7.2).

## Results and discussion

### Significant thermal gradients develop in MEA configurations

**Fig. 2:**
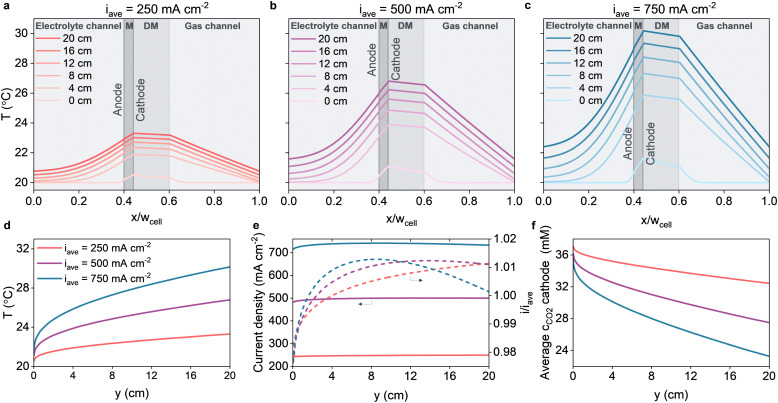
Temperature profiles taken at intervals of 4 cm along the flow direction of the simulated exchange MEA electrolyzers operating at various current densities. The *x*-axis is scaled with the cell width *w*_cell_ which is the width of the configuration excluding the bipolar plate. *M* denotes the membrane, DM denotes the diffusion medium. (a) *ϕ*_applied_ = 2.8625 V, average current density of 250 mA cm^−2^. (b) *ϕ*_applied_ = 2.9725 V, average current density of 500 mA cm^−2^. (c) *ϕ*_applied_ = 3.0325 V, average current density of 750 mA cm^−2^. (d) Evolution of the temperature in the cathode CL along the flow direction. (e) Evolution of the current density along the flow direction. (f) The average CO_2_ concentration in the cathode CL along the flow direction.

Using the expansion method (ESI,† Section 2) it is possible to simulate the two dimensional electrolyzer in the flow-direction. Fig. 2 shows the results for an exchange MEA configuration, where we applied constant potentials along the length, to iteratively calculate the temperature and current density profiles, and arrive at average current densities of 250, 500 and 750 mA cm^−2^ for a down-the-channel length of 20 cm.

Unsurprisingly, higher applied potentials result in higher current densities and larger thermal gradients (Fig. 2d). Considering the chosen channel thickness (0.5 mm), the gradient perpendicular to the electrode is an order of magnitude higher than the gradient along the fluid flow, with a peak temperature in the cathodic catalytic domain (Fig. 2a–c). This peak at the cathode is caused by the higher overpotentials of the COER compared to the OER and the high enthalpy change of carbonate buffering in the cathode (eqn (18) and (19)) compared to the anode. Both contributions completely overshadow the cooling due to the heat of vaporization and the reaction entropy changes within this domain.

In the electrolyte compartment, the thermal boundary layer has not yet penetrated the full width of the channel after 20 cm. Conversely, the thermal boundary layer in the gas compartment already fully develops in the first 4 cm, indicated by the linear temperature profiles within this domain. The faster boundary layer development in the gas phase can be attributed to the lower *Prandtl* in the gas phase compared to the liquid phase, which is approximately 10× as low at ambient conditions. Over the entire simulated length, the current density is relatively constant, indicated by the local current density of 98–102% of the average current density (Fig. 2e). The current density gradually increases with the increasing temperature, due to faster kinetics at higher temperature, until the temperature becomes sufficiently high to cause mass transfer limitations as the solubility of CO_2_ decreases with temperature (Fig. 2f). As the average current density increases, the peak in local current density shifts to an earlier position in the flow cell, because the temperature increases faster. In our study, CO_2_ does not deplete in the bulk gas phase, since a sufficiently high enough gas flow rate has been chosen.

We note that the overall temperature differences across the geometry are relatively mild for the exchange MEA configuration; even at a high current density of 750 mA cm^−2^ the temperature increase is no more than 10 degrees after 20 cm. The anolyte is highly effective in transporting heat from the catalytic domains to the outflow, owing to its superior thermophysical properties of water relative to the gas phase. In practice, the heat transfer to the anolyte is likely affected by bubble-induced convection and local changes in thermophysical properties.^50,51^ The bubble-induced convection could theoretically increase the present heat transfer which would decrease the temperature increase in the exchange MEA electrolyzer. As mentioned above, however, the thermal properties are mainly influenced by the liquid electrolyte, hinting at a lesser importance of bubble formation for the thermal evaluation of an exchange MEA electrolyzer. Therefore, accurately predicting this multiphase phenomena is considered as beyond the scope of this work. In particular, the temperature rise at the walls 
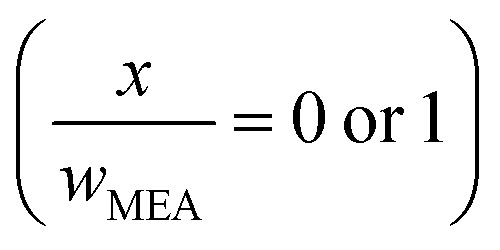
 is relatively mild, due to the high thermal conductivity of the bipolar plates, reflecting a cooling mechanism in the bipolar plates. We also simulated the case without heat exchange to the bipolar plates, *i.e.* a single isolated exchange MEA (ESI,† Section 4). In this case, the bipolar plates and the periodic boundary conditions are omitted. This results in a larger temperature difference between the electrolyte- and gas compartment, peaking to 36 °C at the cathode and gas channel after 20 cm at 3.058 V. In comparison, this is 6 degrees higher than for the case with bipolar plates cooling, which is 60% more temperature rise. Consequently, the isolated exchange MEA reaches a lower average current density of 695 mA cm^−2^, at even slightly higher cell voltage, compared to the periodic case with an average of 750 mA cm^−2^.

In an isothermal large-scale modeling study of a flowing-catholyte configuration performed by Blake *et al.*, a significant decrease in current density was observed along the flow direction.^22^ It was attributed to pH gradients in the catholyte boundary layer, adjacent to the cathodic CL. Our results, using a configuration without catholyte layer, shows a relatively homogeneous current density, which minimizes the energy losses. In addition to the well-known breakthrough problem of flow catholyte configurations,^52^ this nearly constant current density over the entire length suggests that a MEA configuration is more suited for scaling up CO_2_ electrolysis.

**Fig. 3:**
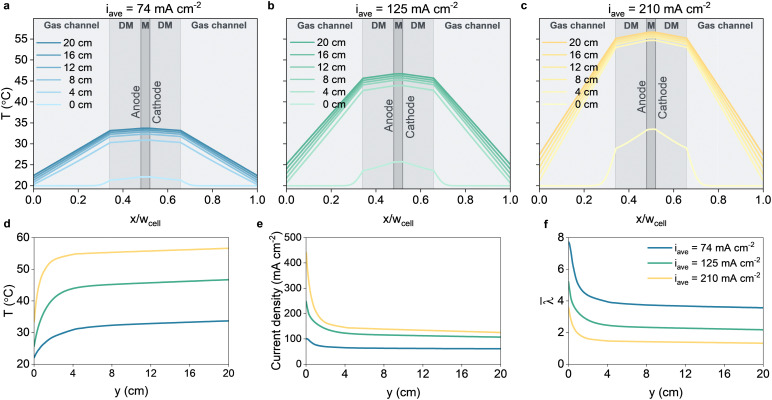
Temperature profiles taken at intervals of 4 cm along the flow direction of the simulated full MEA electrolyzers operating at various current densities. The *x*-axis is scaled with the cell width *w*_cell_ which is the width of the configuration excluding the bipolar plate. M denotes the membrane, DM denotes the diffusion medium. (a) *ϕ*_applied_ = 2.8 V, average current density of 74 mA cm^−2^. (b) *ϕ*_applied_ = 3.05 V, average current density of 125 mA cm^−2^. (c) *ϕ*_applied_ = 3.45 V, average current density of 210 mA cm^−2^. (d) Evolution of the temperature in the cathode CL along the flow direction. (e) Evolution of the current density along the flow direction. (f) The average water content in the membrane along the flow direction.

For the full MEA, the profiles for temperature and current densities completely change (Fig. 3). The temperature profiles show significant thermal gradients, even at relatively low current densities. The temperature sharply increases over the first few centimeters (Fig. 3a–d), accompanied by a sharp reduction in current density (Fig. 3e). This drastic decrease in current density is directly correlated to the dehydration of the membrane, shown in Fig. 3f, which has also been observed in experimental work.^53^ The elevated temperatures in the catalytic domains locally lowers the saturated water vapor pressure thereby allowing water to evaporate from the ionomer phase. The decreased water content lowers the conductivity significantly resulting in high Ohmic losses. While the dehydration phenomena can be observed in 1D models,^20^ its severity is largely underestimated since along-the-channel effects are neglected. Due to the severity of the dehydration, it becomes clear why full MEA configurations for CO_2_ reduction are so difficult to realize.

The membrane dehydration becomes a serious concern in particular for high current densities. At higher current density, the temperature increases faster, which creates a positive feedback loop of faster dehydration (Fig. 3f), which in turn leads to more Ohmic heating and so on. At the highest applied potential (3.45 V cell voltage), the initial current density is over 400 mA cm^−2^, but decreases rapidly below 150 mA cm^−2^ within 3 cm flow height. Further pushing the cell voltage leads to relatively small increase in average current density; a substantial increase in cell voltage from 3.05 V (which is already higher than the highest voltage in the exchange MEA configuration) to 3.45 V, yields only ∼20% higher current density at *y* = 20 cm. The severity of the temperature-induced dehydration in the full MEA configuration is emphasized when realizing that the current density is 3.5× lower than for the exchange MEA, at a substantially higher cell voltage, while already assuming an optimistic case with bipolar plate cooling and relatively small channel length of 20 cm.

**Fig. 4:**
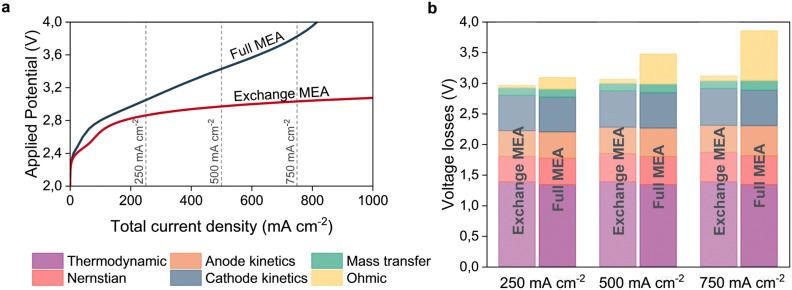
Comparison of the electrochemical performance of the exchange- and full MEA configurations for *y* = 0 operated at ambient conditions. (a) Polarization curves for the two configurations reveal a significantly higher energy requirement for the full MEA. (b) Breakdown of the voltage losses for total current densities of 250, 500 and 750 [mA cm^−2^] which are indicated in figure a with vertical lines.

The significance of the Ohmic losses with respect to the total energy requirements for the full MEA can easily be observed when the energy losses of the two configurations are directly compared. Fig. 4 compares the polarization plots and breaks down the different losses according to the method described by Gerhardt *et al.*^54^ The difference in Ohmic losses becomes increasingly pronounced at higher current densities, while the other contributions only show minor differences. The results shown in Fig. 4 do not consider an expanded domain (*i.e. y* = 0). Therefore, even greater Ohmic losses are expected for along-the-channel results due to the significant decrease in water content as shown in Fig. 3f.

### Higher feed temperatures boost performance of full MEA configuration

**Fig. 5:**
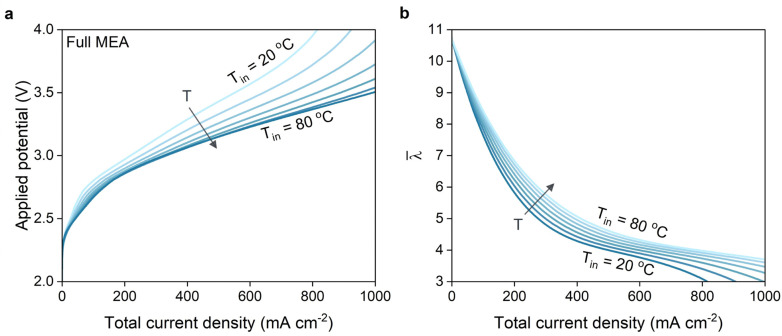
(a) Polarization curves for the simulated full MEA at various operating temperatures ranging from 20 to 80 °C with increments of 10 degrees for *y* = 0. (b) Average membrane water content *versus* the total current density for the same case.

One way of countering membrane dehydration is simply supplying the system with more water. This can be achieved by increasing the temperature of the inlet gases while keeping the gas feed fully humidified, thereby increasing the partial pressure of water in the gas feed. We performed 1D simulations with fully humidified gas inlets at different inlet temperature conditions. The resulting cell voltage and water content (*λ*) for the full MEA configuration are in Fig. 5. The polarization curves in Fig. 5a indicate a significant reduction in power losses, especially at higher current densities where dehydration is particularly prevalent at near ambient conditions. Fig. 5b shows the corresponding average membrane water content which confirms the notion that the membrane is able to retain more water at higher feed temperatures. However, it is likely that dehydration will still be a problem for *y* > 0, which is not considered in the simulations for these results. We expect that significant gradients in the flow direction will still occur based on the results given in Fig. 3 which may lead to even higher temperatures and a higher probability of degradation. An alternative solution would be to periodically inject liquid electrolyte to improve hydration of membranes in full MEA systems.

**Fig. 6:**
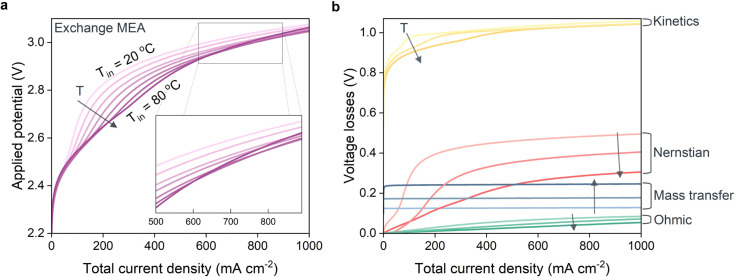
(a) Polarization curves for the simulated exchange MEA at various operating temperatures ranging from 20 to 80 °C with increments of 10 degrees. (b) Voltage breakdown analysis of the major contributions to the voltage losses for the simulated exchange MEA at operating temperatures of 20, 50 and 80 °C.

Dehydration is not an issue for exchange MEA configurations since the ionomer phase is in direct contact with liquid electrolyte at all times. Deviating from ambient temperatures may however still pose advantages. The polarization curves in Fig. 6a show that, dependent on the total current density, different optimum conditions exist. At current densities past 600 mA cm^−2^, the polarization curves of 60 and 70 °C essentially overlap and surpass 80 °C in terms of electrochemical performance. This agrees with the experimental observations by Löwe *et al.* who showed that the optimum performance is not obtained at the highest operating temperature.^18^

To elucidate the phenomena causing an optimum at 60–70 °C, we break down the energy losses again to differentiate between the kinetic, Nernstian, mass transfer and Ohmic losses for operating temperatures of 20, 50 and 80 °C, up to 1000 mA cm^−2^ (Fig. 6b).^54^ Unsurprisingly, for voltage losses due to reaction kinetics and Ohmic contributions decrease at higher operating temperatures. The higher losses due to CO_2_ mass transfer limitations reflect the reduced solubility of CO_2_ at higher temperatures. The Nernstian- and Kinetic losses are more complex and have intersecting curves. That is because both terms are temperature dependent, but also highly dependent on the local OH^−^ concentration within the catalytic domains. The OH^−^ concentration varies with temperature due to the temperature-dependence of the diffusion, but particularly because of slower water dissociation rates at higher temperatures. Overall, this results in milder pH gradients which reduce the Nernstian losses. The breakdown analysis also reveals the origin of the bend in the polarization curves for the exchange MEA around 100 mA cm^−2^. It can be attributed to the kinetic- and Nernstian losses, both of which are functions of the local pH, which undergoes a large shift in the catalytic domains as carbonate buffering becomes less effective at higher current densities.

### Parasitic hydrogen evolution reaction in the full MEA configuration enhances dehydration

The faradaic efficiency (FE) of CO_2_ conversion is an important metric for determining its feasibility. Low FE_COER_ can be a consequence of the contamination of the catalytic domain with metallic impurities. For example, work by Won *et al.* found that iron impurities decreased the FE_COER_ from 80% to 20% in a matter of 30 minutes for the electrochemical reduction of CO_2_.^55^ As opposed to when COER and OER are prevalent (eqn (2) and (3)), occurrence of HER and OER (eqn (2) and (4)) will result in net water consumption. Combined with our observation that dehydration is already a significant problem for the full MEA configuration (Fig. 3), it is to be expected that a poor FE towards the COER will result in amplified dehydration.

**Fig. 7:**
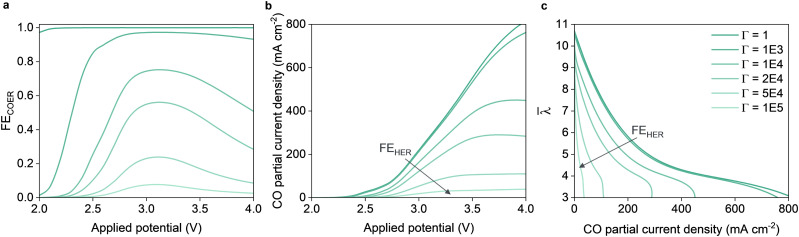
Electrochemical performance of a full MEA simulated with increased HER kinetics. (a) Faradaic efficiency of the carbon monoxide evolution reaction at various potential for different degrees of increased HER kinetics (*Γ*). (b) Polarization curves and (c) average membrane water content for the various degrees of HER amplification.

In order to illustrate this likely problem, the kinetic parameters of the HER were manually altered by multiplying the exchange current density of the HER with a factor *Γ* [−], reflecting the case of metallic impurities with a high affinity towards HER. *Γ* is varied between 1 and 100 000 which results in a FE_COER_ varying between 0 and 1 as can be seen in Fig. 7a. In case of the unmodified kinetic parameters (*Γ* = 1), FE_COER_ is 1 for almost the entire applied potential range. Fig. 7b shows the applied potential plotted against the partial current density towards the COER. With decreased FE_COER_, the required energy to synthesize CO increases significantly. The energy per produced CO increases rapidly both because the supplied energy is now channeled towards the HER, and partly because the Ohmic resistance increases due to the rapid membrane dehydration as shown in Fig. 7c.

### Evaluating the heat generation mechanisms

**Fig. 8:**
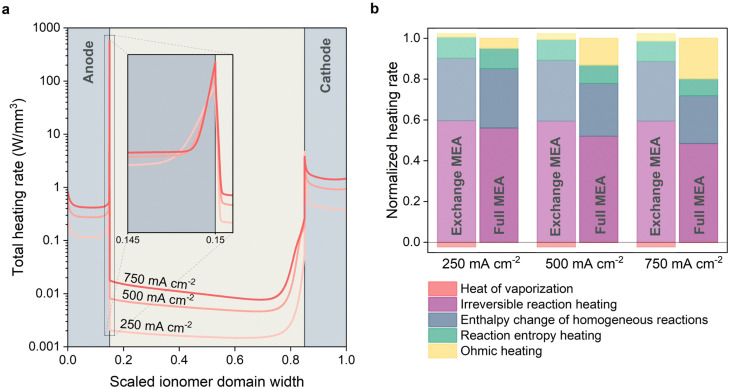
(a) The heating profile over the catalytic domains and the membrane for the exchange MEA indicate that the largest heating mechanisms occur within the catalytic domains. For clarity, the width of the domain has been scaled such that *W*_CL_ : *W*_M_ is 0.15 : 0.7 while in the model it is 0.1 : 1 Heating contributions in the full- and exchange MEA configurations normalized with the total heating rate for 250, 500 and 750 mA cm^−2^. (b) Heating contributions in the full- and exchange MEA configurations normalized with the total heating rate for 250, 500 and 750 mA cm^−2^.

In order to gain further insights into the heating phenomena and guide future simulation work, it is worthwile to breakdown the various heating generation terms. For this analysis, we focus on the heat generation in the catalytic layers and the membrane, since the majority of the heat is generated here. Absolute heating profiles for the exchange MEA given in Fig. 8a reveal that most thermal energy is generated in the catalyst layers, especially at the interface between the catalyst layer and the membrane (see inset). Although it is not surprising to observe a peak in heating rate at the interface where the electrochemical activity is concentrated, and in agreement with previous modeling work,^21^ the volumetric heating rate is an impressive 10–1000× larger at this interface compared to the rest of the catalytic domain. The heating profiles also suggest that the heating rate within the anodic catalyst layer is more significant, at most 150× higher at 750 mA cm^−2^. While the overpotential for the COER compared to that of the OER is higher, substantial cooling occurs within the cathodic catalytic domain as the reaction entropy term is negative resulting in a lower overall heating rate compared to the anodic catalytic domain.

The heating rates in the considered domains are integrated and normalized with the total heating rate to yield the normalized heating contributions which are plotted for three current densities (Fig. 8b). For the full MEA, the increased resistance due to a reduced water content is represented by a significant increase of the Ohmic heating contribution. At a current density of 250 mA cm^−2^ at *y* = 0, the contribution is only 6% which increases to 25% at 750 mA cm^−2^. We can expect, based on the 2D results in Fig. 3, that the Ohmic losses are increasingly important for the full MEA when progressing further down the channel.

Besides the Ohmic heating, most heat is released in the form of irreversible reaction losses (*i.e.*, reaction overpotentials), followed by heat associated to the enthalpy changes of the homogeneous reactions (such as carbonate buffering) and reaction entropy changes. Homogeneous reactions are sometimes omitted in models for simplicity, however as our models suggests, their high reaction rates in the catalytic domains play an integral role in the generation of heat. Additionally, their effect on the composition of the local micro-environment has a significant effect on the physical phenomena within the catalytic domains and they should therefore be included. The heating contribution due to the evaporation of water is insignificant for the full MEA configuration as the membrane water content is in equilibrium with the gaseous water content at steady state.

The normalized contribution for the exchange MEA shows a similar heating distribution whereby the most prevalent heating is because of irreversible reaction losses, examplifying the poor kinetics associated with electrochemical CO_2_ reduction. The ranking is followed by heating due to homogeneous reactions and entropy changes. Ohmic heating also shows an increasing contribution due to the quadratic nature of resistance, but is of a much lesser importance compared to the full MEA configuration as dehydration is insignificant. The continuous evaporation of water from the ionomer phase to the gas channel at the cathode side results in a cooling contribution which increases with current density. This can be attributed to the larger temperature gradients at higher current densities, leading to an increase in the local saturation vapor pressure which increases the driving forces for evaporation.

## Conclusions

In this work, we developed a non-isothermal model capable of predicting large-scale 2D effects for the electrolysis of CO_2_ into CO using a MEA configuration. We observed that the majority of heat is produced within the catalytic domains because of the high overpotentials associated with the kinetics- and entropy changes of the charge transfer reactions as well as the homogeneous reactions such as carbonate buffering. Ohmic heating plays a major role in full MEA configurations since dehydration of the membrane greatly increases the resistance of the ionomer phase. This adverse effect becomes increasingly pronounced at higher current densities or when the faradaic efficiency for CO_2_ conversion decreases. Our two-dimensional model demonstrates significant thermal gradients, from 20 °C to 55 °C in 20 cm flow length, at an average current density of 210 mA cm^−2^. The dehydration and thermal gradients induce poor electrochemical performance. By operating at elevated temperatures, membrane dehydration can theoretically be mitigated as this allows for more water to be delivered to the system. Unfortunately, this only delays inevitable dehydration-related problems which suggests that full MEA configurations are not suitable for scale-up.

In contrast, our simulations of the exchange MEA configurations indicate minor thermal gradients which have little to no effect on the electrochemical performance along the channel, indicating promising results for scale-up. The high performance is attributed to the high heat transfer rates of the anolyte as well as the superior mass transport of liquid-equilibrated membranes. The performance of an exchange MEA configuration can be further improved by tuning the feed temperature. In that case, a trade-off establishes between faster kinetics, higher ionic conductivity and smaller pH gradients at one hand, and lower CO_2_ solubility at the other hand, which predicts an optimum feed temperature between 60–70 °C.

## Data availability

The complete models (as.mph COMSOL files), for both the exchange MEA and full MEA, including the model result and readme file, are available at Zenodo, with DOI https://doi.org/10.5281/zenodo.13379477. Additional results are available in the ESI.† This contains details about model expansions and validations, computational details and boundary conditions, parameters and additional equations, and temperature distributions for isolated MEAs.

## Conflicts of interest

There are no conflicts to declare.

## Supplementary Material

EY-003-D4EY00190G-s001
