# Supplementary material for: Heating dictates the scalability of CO2 electrolyzer types
Source: EES Catal. 2024 Dec 27;3(2):305–17. doi: 10.1039/d4ey00190g (PMC11721209; doi:10.1039/d4ey00190g)
Supplement: EY-003-D4EY00190G-s001 [file EY-003-D4EY00190G-s001.pdf]

Supplementary Information (SI) for EES Catalysis.
This journal is © The Royal Society of Chemistry 2024

Supplementary Information

for

Heating Dictates the Scalability of CO_2 Electrolyzer Types

Jan-Willem Hurkmans[1], Henri M. Pelzer[1], Tom Burdyny[1], Jurriaan Peeters[2], David A. Vermaas[1*]

[1] Department of Chemical Engineering, Delft University of Technology, 2629 HZ Delft, The Netherlands

[2] Process & Energy Department, Delft University of Technology, 2628 CB Delft, Netherlands

Contents

1. Model Comparison and Validation 1
2. Expansion Method Approach and Validation 3
3. Artificial Charge Separation Limiter 5
4. Temperature Distributions for an Isolated MEA 9
5. Computational Set-up and Meshing 10
6. Boundary Conditions 12
7. Additional Governing Equations, Calculations for Parameters and Variables 14
8. Simulation Parameters 20
9. Bibliography 22

1 Model Comparison and Validation

The model developed in this work is validated against the seminal modeling work by Weng et al and the experimental work by Hansen et al [1, 2]. The polarization curves of the exchange MEA operated at ambient conditions for this work and the validation cases are given in **Figure S1**.

The model developed by Weng et al considers both the Exchange MEA and the Full MEA configuration in a one-dimensional system. The model developed in this work is based on the framework proposed by Weng, but contains crucial deviations. Firstly, we consider a two dimensional system which has influence on the boundaries of the model which will vary along the flow direction. Secondly, the computational domain in this work includes the flow compartment at the anode side of the MEA configurations. This has influence on the boundary conditions for the species- and water transport. For example, this allows for the implementation of a source term for the water content at the anode side for the Full MEA simulations. This introduces a more accurate description of membrane equilibration compared to setting a Dirichlet boundary condition with a constant value for λ. Regarding heat transfer, two main differences can be found. Firstly, the source term regarding heat generated due to homogeneous chemical reactions in our work has a negative sign (i.e. $Q_{HR} = -\sum_k \Delta H_k R_{k,HR}$) in order to conform to the endothermic and exothermic nature of the reactions based on their enthalpy change. Secondly, Weng et al implement a temperature dependence on the equilibrium constant for the homogeneous reactions but neglect the temperature dependence of the respective reaction rates, resulting in rate constants which are inconsistent with the rates measured experimentally by Schulz et al [3]. In general, the two plots follow a similar trend whereby our work reports a slightly worse electrochemical performance. Interestingly, in the low current density range a dip can be found in our results, which is a direct consequence of the concentration of dissolved species in the catalytic domains as seen in **Figure 6b** in the main text.

For the validation, the work by Hansen et al was chosen since the system closely resembles the system modeled in this work. Most importantly the work of Hansen et al. uses a CCM on the anode as well as cathode side. Ag is used as a reduction catalyst at the cathode whereas IrO_2 is used as an anode side catalyst. The use of a hot pressing method greatly reduces the interfacial resistance between the catalytic layer and the membrane which is a resistance typically not included in models. Thus, the setup used by Hansen et al. poses the closest comparable setup present in current literature. Next to the CCM (using Orion AMX 2.8) Hansen et al use a CO_2-saturated recirculating 0.01 M $KHCO_3$ anolyte. Plain carbon felts are used as a diffusion layer on the cathode side while a platinized titanium felt acts as a diffusion layer on the anode side. The full cell is assembled in a zero-gap (exchange MEA) configuration. In addition to Hansen's work, several experimental works are mentioned in Table S1 including different current density ranges and anolyte concentrations as well as anolyte types. Only experimental work that includes current densities of at least 400 mA/cm^2 is considered.

When comparing the voltage of the model in this work to the experimental results displayed in Table S1, we note that the voltages in the experimental results of refs [2] and [4] are close to the model results for a hybrid MEA. However, in particular for refs [5-8], the experimental cell voltages exceed the voltages calculated by the model in all cases, especially at higher current densities. This can be accounted to the contact resistance present in the experimental setups, adding a higher ohmic resistance, which becomes more striking at higher current densities. In this model, the overall ohmic resistance is minor, due to neglected contact resistance, which would be present if the CCM method is used. To the best of the authors' knowledge, Hansen et al. is the only present work up to date utilizing CCM and thus minimizing contact resistance. Therefore the comparison to the data displayed in Figure S1 remains the most telling experimental data the model can be compared to. To make the model results comparable to the results in Table S1, contact resistances would need to be added. However, the increased ohmic resistance would lead to a higher level of dissipated heat. This shows that the model displayed in this work can be seen as a minimal 'ideal' case for a scaled-up CO_2 electrolyzer which underlines the bottleneck the heat generation of the device will impose on the system.

Table S1: Overview of experiments with high current densities (>400 mA/cm^2). The displayed results are generally higher than the results calculated by the model in this work due to the higher ohmic resistance due to the present contact resistance in the

*experiments displayed. *Work by Hansen et al. taken as comparison to the model in this work as CCM is used **Dynamic operation by periodically injecting highly concentrated or KOH or CsOH solution.*

Cell Configuration	Catalyst (Cathode/Anode)	Membrane	Electrolyte	Current [mA/cm²]	Voltage [V]	FE_{CO} [%]	Ref.
MEA (CCM)*	Ag/IrO_2	Orion AMX 2.8)	0.01 M KHCO3	30-800	2.7-3.3	>95	[2]
MEA	Au/IrO_2	QAPPT	APE/Water	10-500	1.86-3	>80	[4]
MEA (Stack)	Ag/IrO_2	Sustainion (X37-50 Grade RT)	0.1 M CsHCO3	100-400	2.8-3.3	>90	[5]
MEA	Ag/IrO_2	PiperIon	0.1 M CsOH	10-1000	2.6-3.4	Up to 90	[6]
MEA**	Ag/IrO_2	Sustainion (X37-50 Grade RT)	Water	350-850	3.0-3.5	Up to 70	[7]
MEA	Cu/IrO_2	QAPPT	0.1 KHCO3	10-600	2.3-3.7	NA	[8]

Comparing the model results to the experimental data of Hansen et al., we observe that up to around 400 [mA cm^{-2}], the experimental work matches the simulated results decently. This indicates that the model covers the equilibrium potential and the kinetic overpotential governing at lower current densities and thus lower potentials accurately. At larger applied potentials, the Ohmic losses in the experimental set-up start to differ from the model which can be attributed to various phenomena not incorporated in our model. For instance, remaining, partly nonlinear, contact resistances, bubble formation at the anode catalyst layer, salt deposition, non-ideal membrane behavior, and three-dimensional land-channel effects. These effects become more severe at higher current densities explaining the increasing difference from model to experimental data. However, the model underestimates the voltage and hence the dissipated heat, which shows that the temperature in experimental scalable setups might increase even more severely than predicted by the model at higher current densities even if CCM are used.

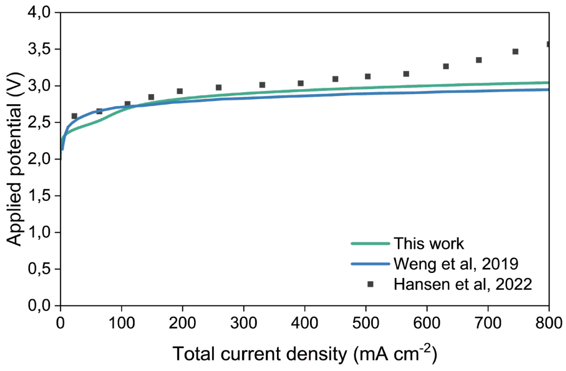

Figure S1 *Polarization curves of experimental work by Hansen et al [2], a comparison to the modeling work by Weng et al [1] and the results obtained in this work for an exchange MEA operated at ambient conditions.*

2 Expansion Method Approach and Validation

The model developed in this work is computationally challenging to solve. Especially the inclusion of the homogeneous reactions in liquid and ionomer domains fully coupled to the local ionic conductivity imposes convergence problems. In order to reach convergence, an extremely fine mesh is required, especially near the interface of the catalytic domains and the membrane. Reaching convergence in one dimensional computational domains is already difficult to achieve at higher applied potentials. So in order to be able to predict large scale behavior, a simplification is required.

Since it is possible to predict short lengths along the flow direction (<0.1 mm), we can exploit the fact that the gradients (for all solved variables) in the x-direction within the MEA domain greatly exceed the gradients in the y-direction, i.e. the transport here is essentially one dimensional. Additionally, the convective transport in the direction of the flow is much higher compared to diffusional transport which means we can neglect back diffusion (This is not particularly true for the diffusion of heat, however the validation in this section indicates that this is relatively unimportant). These conditions allow us to decompose the total length of the domain of interest into small sub cells which are solved sequentially. This approach was previously used by Blake et al [9], however our approach uses much smaller sub-cells since our non-isothermal model includes more electrolyzer domains and the governing equations have a greater complexity. This expansion method is also comparable to 1+2D approaches which have been implemented for fuel cell models [10].

A schematic representation of the expansion method is depicted in **Figure S2**. Initially, sub cell 0 is solved with a desired applied potential. The solution at the half way point of the sub cell is then used as inlet conditions for the next cell until the complete length of the domain is resolved. Mathematically, the inlet conditions for each cell can be expressed as:

$$N_i(x, y = 0) = N_{i-1}\left(x, y = \frac{1}{2}L_{cell}\right)$$

Whereby N_i is the field of a certain variable (i.e. temperature or concentration) for the ith cell. The half way point is chosen since it is largely unaffected by the no-flux boundary conditions set at the outlets ($y = L_{cell}$). When the subsequent simulations have finished, it is possible to reconstruct the solution to the entire computational domain.

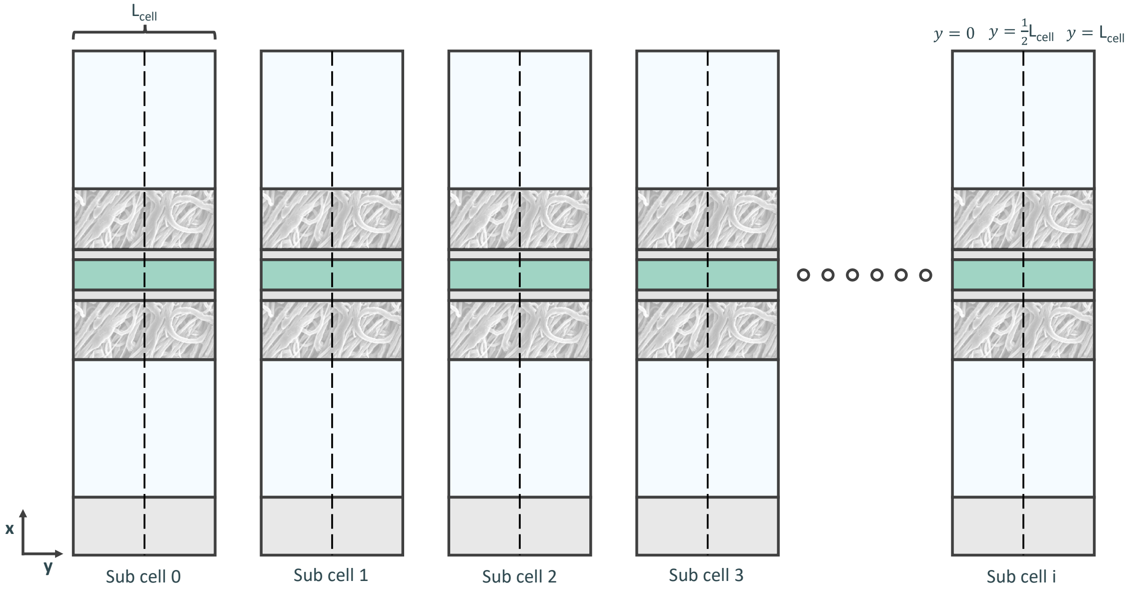

Figure S2 *Schematic depiction of the expansion method used in this work. The sub cells are solved subsequently whereby the inlet (y=0) and initial conditions of a cell i are based on the solutions of the previously solved cell (i-1).*

Since the dimensions of the sub cells in this work are very small, the question arises whether it is valid to use this decomposition method. In order to test this, and to validate the method, a simple test was set up which included solving the temperature field of a simplified MEA configuration with a total length of 2 cm via two ways; i) the entire domain is solved at once, ii) the domain is subdivided into sub cells with a width of 0.1 mm resulting in 400 subsequent simulations.

The simplified model only includes transport of gases, fluid flow and heat transfer as described in the main body of the text. In the catalytic domains, a constant heat source of $10^{10}[\mathrm{W\ m^{-3}}]$ and a constant mass source of 1000 $[\mathrm{kg\ m^{-3}\ s^{-1}}]$ CO are chosen arbitrarily. In order to assess the difference, the average temperature in the cathode catalytic layer is plotted against the flow direction, the results of this test are shown in **Figure S3**. The expansion method slightly underestimates the temperature since part of the heat presumably diffuses in the flow direction. However, the error is 0.4% at most and it decreases along the flow direction down to 0.055% at y = 2 cm. Consequently, the method is deemed reasonable for the purpose of this work.

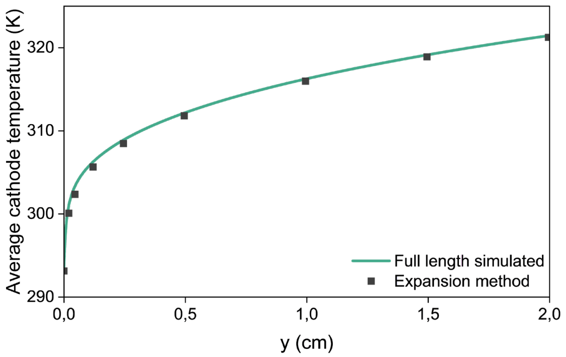

Figure S3 *Validation of the expansion method. The continuous line represents the simplified simulation of a domain with a total length of 2 cm. The circles represent the reconstruction of the temperature distribution computed with the expansion method taken at a few arbitrary coordinates along the flow direction. In this case, the entire length is subdivided into sub cells with a length of 0.1 mm. This corresponds to 400 subsequent simulations. The largest error is 0.4%, which is recorded in the first few mm. The error at 2 cm is 0.055%.*

3 Artificial Charge Separation Limiter

The conservation of ions in aqueous media (including membranes), is modeled using the Nernst-Planck equation. For a species *i*:

$$\frac{\partial c_i}{\partial t} + \nabla \cdot \boldsymbol{J_i} = R_i \quad \text{(S1)}$$

Whereby the total flux can be expressed as:

$$\boldsymbol{J_i} = -D_{i,eff} \nabla c_i + \boldsymbol{u_l} c_i - D_{i,eff} \frac{z_i F}{RT} c_i \nabla \phi_l \quad \text{(S2)}$$

Here the first term describes diffusion, the second term describes convection and the third term describes electromigration. The conservation equation can be solved simultaneously with charge conservation laws to resolve concentration fields and other electrochemically relevant scalar- and vector quantities. For length scales above the micron scale, the tertiary current distribution is arguably the most accurate numerical approach. An important part of the framework is the assumption that electroneutrality is valid for a given region of space, i.e. this domain must always contain the same number of charges of either sign. Simply solving for all the species in a system will not grant electroneutrality automatically, therefore the approach commonly used is to solve for *n-1* species in a system with *n* species. The remaining species (denoted below as species *j*) is distributed according to the local charge imbalance such that electroneutrality is ensured everywhere:

$$c_j = -\frac{\rho_{fix}}{z_j F} - \frac{\sum_{i=1}^{n-1} z_i c_i}{z_j} \quad \text{(S3)}$$

Here, the first term represents the fixed back charge density in case of an ionic exchange membrane system. This method is often called the *tertiary current distribution.* The species c_j $[\mathrm{mol\ m^{-3}}]$ should ideally be an inert species and abundantly present in the system to ensure numerical stability and physically sound results [11].

When modeling electrochemical systems with an effectively unlimited amount of electrolyte (as is the case when an exchange solution is used), this approach is typically unproblematic. However, problems arise when a closed system is employed wherein the inert species is not abundant. An example of such a system is the full-MEA architecture. Here, the system is limited to a fixed amount of charge and more importantly, a fixed amount of inert species. An example is given below, in **Figure S4**. Here, sufficiently large concentration gradients appear. When equation S3 is employed to distribute species K^+ in order to make sure electroneutrality is valid everywhere, it becomes apparent that unphysical negative concentrations appear as shown in **Figure S5**.

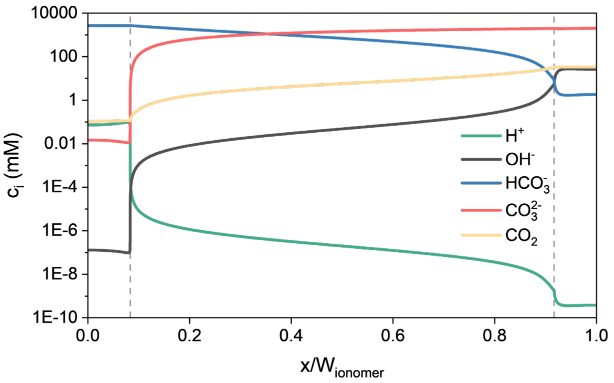

Figure S4 *Concentration profiles of H^+, OH^-, HCO^-_3, CO^{2-}_3, and CO_2 dissolved in the ionomer phase of a Full MEA configuration at operation at approximately 200 $[mA\ cm^{-2}]$ obtained using the tertiary current distribution. The x-axis has been scaled with the width of the ionomer domain ($W_{ionomer} = 2W_{CL} + W_M$). The interface between the catalytic layers and the membrane are denoted with dashed lines. The corresponding K^+ concentration can be found in **Figure S5.***

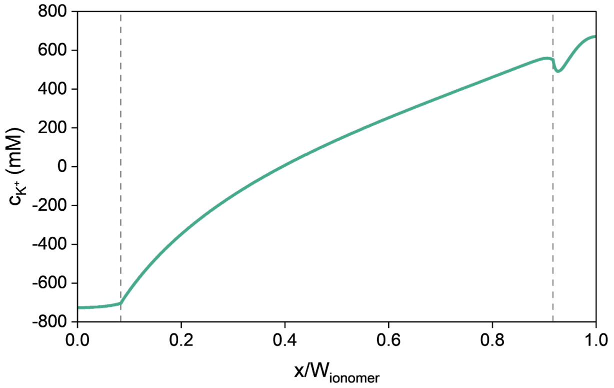

Figure S5 *Concentration of K^+ in the ionomer phase during operation at approximately 200 $[mA\ cm^{-2}]$. The x-axis has been scaled with the width of the ionomer domain ($W_{ionomer} = 2W_{CL} + W_M$). The interface between the catalytic layers and the membrane are denoted with dashed lines. The concentration has been determined with the tertiary current distribution based on the concentration distribution given in **Figure S4**, which results in negative concentrations in order to achieve electroneutrality throughout the ionomer phase.*

Alternatively, electroneutrality can be neglected and all *n* species can be solved for. This results in positive concentrations everywhere, however electroneutrality is in this case not valid as charge separation occurs simply due to diffusion and migration. This is also not physically accurate since very intense electric fields will be required for charge to separate [12].

Concluding on these two methods:

- Using a *tertiary current distribution* approach results in an electroneutral membrane. This however results in negative concentrations of K^+ in the membrane.
- Neglecting the electroneutrality constraint does not result in negative concentrations, but leads to a membrane with separation of charges.

It becomes apparent that another method is required to in order to ensure electroneutrality and prevent negative concentrations. An obvious, strictly accurate approach would be to solve the Nernst-Planck-Poisson equations

which will result in a fully resolved charge distribution including the double layers. This method is however computationally very costly for even relatively small domains. An alternative approach is proposed here based on a simple notion that a local excess of charge leads to negligible concentration gradients but to a very high electric field strength [13]. This means that charge separation is essentially hindered by migration in local potential gradients. In this approach, the important difference with the *tertiary current distribution* is that all the charged species act on charge separation proportional to their mobility and valence. The effect is artificially introduced by adding an artificial flux to the Nernst-Planck equation which is driven by charge gradients. The total flux is now expressed as:

$$\boldsymbol{J_i} = -D_i \nabla c_i + \boldsymbol{u_l} c_i - D_{i,eff} \frac{z_{i,eff} F}{RT} c_i \nabla \phi_l \tag{S4}$$

Wherein the effective valence, $z_{i,eff}$, embeds the *Artificial Charge Separation Limiter* (ACSL):

$$z_{i,eff} = z_i \left(1 - D_q \frac{\max(|q_{loc} + q_{fix}|)}{\nabla \phi_l} \nabla q_{loc}\right) \tag{S5}$$

Whereby q_{fix} $[\mathrm{C\ m^{-3}}]$ is the charge density of the background charge and q_{loc} is the local charge density ($q_{loc} = F \sum_{i=1}^{n} z_i c_i$). As can be seen from equation S5, the limiter vanishes when global electroneutrality is ensured ($q_{loc} = q_{fix}$). The artificial charge diffusion coefficient D_q has units $[\mathrm{V\ m^6\ C^{-2}}]$ in order to conform to the non-dimensionality of the valence. Its value is chosen through trial and error to ensure numerical stability. Within the simulations in this work it is kept between 0.1-100.

Below, in **Figure S6** and **S7** several results will be shown which compares the use with- and without the ACSL. It is important to keep in mind that while the idea behind the limiter has a physical origin, the implementation is not. It is therefore encouraged that approaches based on physical argumentation are developed.

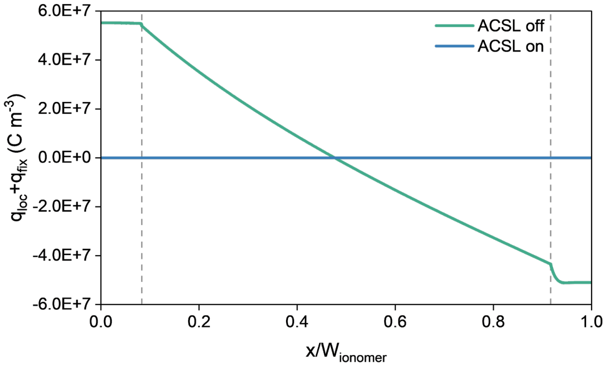

Figure S6 *Showcase of the effectiveness of the ACSL. When the ACSL is used (blue line), the charge density (The sum of the fixed back charge and the mobile ionic charge) is zero throughout the ionomer phase. The x-axis has been scaled with the width of the ionomer domain ($W_{ionomer} = 2W_{CL} + W_M$). The interface between the catalytic layers and the membrane are denoted with dashed lines.*

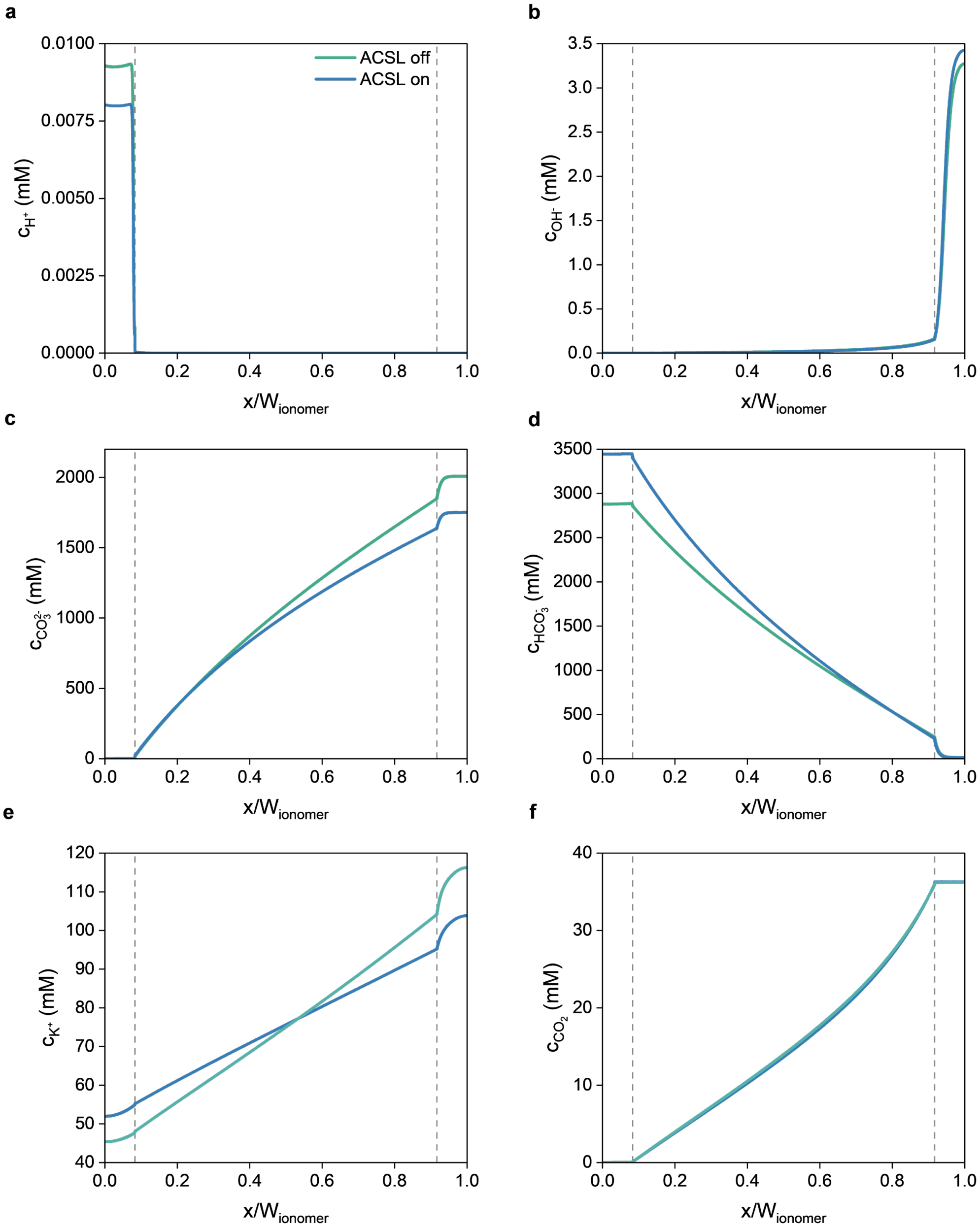

***Figure S7** Comparison of concentration profiles of dissolved species in the ionomer phase when the ACSL is employed. Blue line corresponds to ACSL = on. Green line corresponds to ACSL = off. The x-axis has been scaled with the width of the ionomer domain ($W_{ionomer} = 2W_{CL} + W_M$). The interface between the catalytic layers and the membrane are denoted with dashed lines. Concentration profiles of **a)** H^+ **b)** OH^- **c)** CO_3^{2-} **d)** HCO_3^- **e)** K^+ and **f)** CO_2*

4 Temperature Distributions for an Isolated MEA

In this modeling work, it has been chosen to employ periodic boundary conditions for the temperature field in order to mimic an electrolyzer in a scaled up (i.e. stacked) setting. Here, we present the case of a fully isolated configuration in order to highlight the difference. For this case, an exchange MEA operated at an applied potential of 3.058 [V] is simulated according to the same method used to generate the results presented in **Figure 2**, with the only difference that the bipolar plates and the periodic boundary conditions are omitted (i.e. no flux boundary conditions are used at the $x/w_{MEA} = 0$ and $x/w_{MEA} = 1$).

The results for this simulation are given in **Figure S8**. The average current density of 695 $[mA\ cm^{-2}]$ is relatively close to the results of **Figure 2c**, whereby an average current density of 750 $[mA\ cm^{-2}]$ is recorded. A comparison between the two cases indicates a clear difference. Firstly, the isolated case has a clear temperature difference between the electrolyte channel and the gas channel, which is not observed for the periodic boundary case as periodic boundary homogenizes the heat distribution. The large discrepancy between the two channels can again be attributed to the lower *Prandtl* number in the gas phase; the relatively fast boundary layer development leads to full penetration over the gas channel width at which point the heat transfer to the gas becomes increasingly worse leading to a rising overall temperature in the domain. This also results in a clear quantitative difference; at 20 cm the peak temperature reaches nearly 36 ^{o}C, as opposed to approximately 30 ^{o}C for the periodic case, which exhibits a lower current density. The higher temperature also results in a larger variation in the current density along the flow channel (**Figure S8b**).

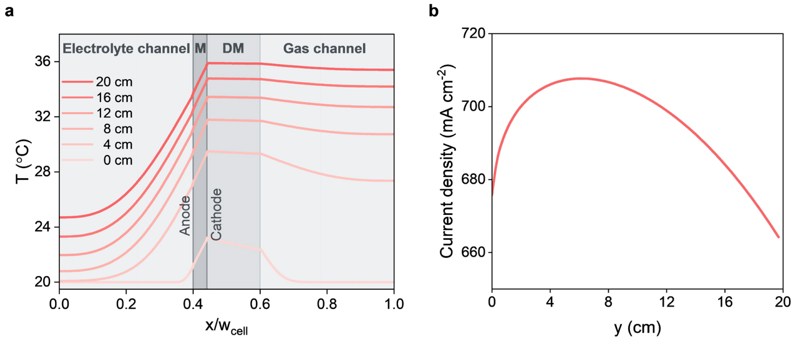

Figure S8 a) *Temperature profiles taken at intervals of 4 cm along the flow direction of a simulated fully isolated Exchange MEA electrolyzer operating at an applied potential of* 3.058 $[V]$*. The x-axis is scaled with the cell width* w_{cell} which is the width of the configuration with *the bipolar plate omitted. M denotes the membrane, DM denotes the diffusion medium.* ***b)*** *Evolution of the current density along the flow direction, the average current density is* 695 $[mA\ cm^{-2}]$.

5 Computational Set-up and Meshing

The model was built in COMSOL Multiphysics v5.6 and solved with a relative tolerance of 0.001. An overview of the meshes used to run the base models are given in **Tables S1** and **S2**. In general, meshing within the ionomer domains (the catalytic layer and the membrane) requires a very high mesh density in the x-direction due to the homogeneous reactions. Refinement near the interface between the catalytic layers and the membrane is essential to reach convergence at higher applied potentials.

Mesh dependency was analyzed for the simulations, with a specific example provided for the Exchange MEA. A simulation at ambient conditions was performed for an applied potential range of 2–3 V. The number of mesh cells in the x-direction within the ionomer domains was varied between 44 and 1760, while the solving method remained consistent. To assess mesh independence, the total current density was plotted, revealing similar trends for other metrics, such as the average membrane temperature. The results are illustrated in **Figure S9**. At low potentials, meshes with fewer than 200 cells fail to converge at 2.5 V. At higher potentials, the findings highlight an optimal mesh density, as most simulations struggle to reach 3 V.

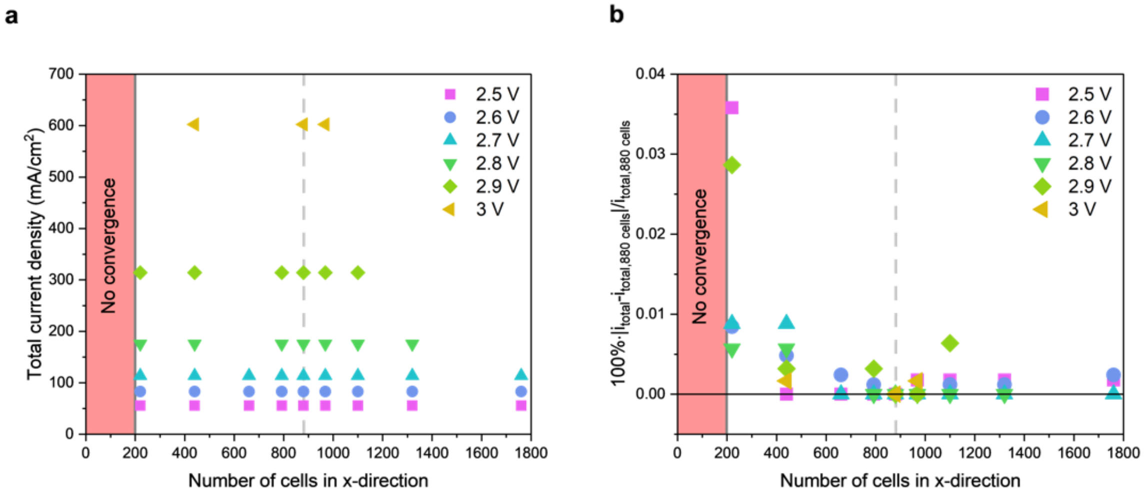

Figure S9 *Mesh dependency study for the Exchange MEA configuration. The simulation was found to be very sensitive to the number of cells in the domains where homogeneous reactions occur where gradients are in the x-direction. Therefore, the amount of cells in the x-direction was varied between 40 and 1480 and the Exchange MEA model was ran from 2-3 V. Here, the total current density is plotted to indicate the mesh independency. The dashed line indicates the mesh density used in this study.* ***a)*** *Total current density as a function of number of cells in ionomer domain in the x-direction show the difficulty of convergence at higher applied potentials.* ***c)*** *Percentual difference with the chosen cell density of 880 shows very little mesh dependency.*

Table S2 *Full MEA mesh details. The domains are schematically depicted (not to scale) in* ***Figure S10****.*

Domain	Meshing type	Dimensions x-direction	Dimensions y-direction	Refinement
**Gas channels (a,g)**	Triangular	Min: 3E-8 [m]<br>Max: 0.7E-5 [m]	Min: 3E-8 [m]<br>Max: 0.7E-5 [m]	-
**Diffusion media (b,f)**	Rectangular	70 mesh elements	6 mesh elements per 10 micron	Up to 50 micron
**Catalytic layers (c,e)**	Rectangular	150 mesh elements	6 mesh elements per 10 micron	Up to 1 nm
**Membrane (d)**	Rectangular	1000 mesh elements	6 mesh elements per 10 micron	Up to 1 nm
**Bipolar plate (h)**	Rectangular	50 mesh elements	6 mesh elements per 10 micron	Up to 1 micron

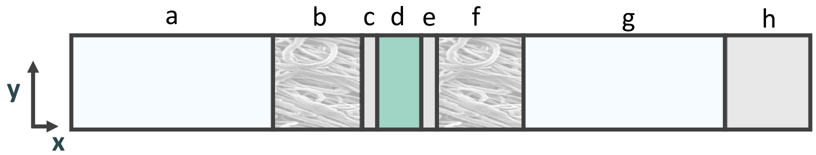

Figure S10 *Overview of meshed domains of the Full MEA. Identification corresponds to* ***Table S1****.*

Table S3 *Exchange MEA mesh details. The domains are schematically depicted (not to scale) in* ***Figure S11****.*

Domain	Meshing type	Size x-direction	Size in y-direction	Refinement
**Anolyte channel (a)**	Triangular	Min: 2.5E-8 [m]<br>Max: 1.5E-6 [m]	Min: 2.5E-8 [m]<br>Max: 1.5E-6 [m]	-
**Gas channel (g)**	Triangular	Min: 2.5E-8 [m]<br>Max: 0.5E-5 [m]	Min: 2.5E-8 [m]<br>Max: 0.5E-5 [m]	-
**Anolyte channel refinement (b), width = 5 micron**	Rectangular	80 mesh elements	6 mesh elements per 10 micron	Up to 25 nm
**Diffusion medium (f)**	Rectangular	120 mesh elements	6 mesh elements per 10 micron	Up to 20 micron
**Catalytic layers (c,e)**	Rectangular	140 mesh elements	6 mesh elements per 10 micron	Up to 1 nm
**Membrane (d)**	Rectangular	600 mesh elements	6 mesh elements per 10 micron	Up to 1 nm
**Bipolar plate (h)**	Rectangular	50 mesh elements	6 mesh elements per 10 micron	Up to 1 micron

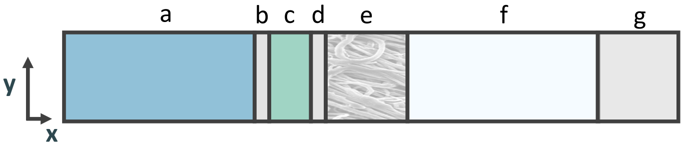

Figure S11 *Overview of meshed domains of the Exchange MEA. Identification corresponds to* ***Table S2****.*

6 Boundary Conditions

A detailed overview of the boundary conditions is given in **Tables S3** and **S4**. Note that for the expansion simulations used to generate the results in **Figures 2** and **3** (main text) use different boundary conditions for boundaries 2-9 (Full MEA) and 2-8 (Exchange MEA). Details regarding this approach can be found in section 2.

Table S3 *Overview of boundary conditions used in simulations for the Full MEA configuration. A schematic overview of the corresponding boundaries is given in* ***Figure S12****. *index refers to the number of the sub cell in the expansion method.*

Boundary	Potential distribution	Water transport	Dissolved species transport	Darcy's law	Transport of gaseous species	Heat transfer
1	-	-	-	-	$no\ flux$	$Periodic$
2	-	-	-	-	$\omega_i = \omega_{i,in}$	$T = T_{op}$
3	$no\ flux$	-	-	$-\boldsymbol{n} \cdot \boldsymbol{u} = 0$	$no\ flux$	$no\ flux$
4	$no\ flux$	$no\ flux$	$no\ flux$	$-\boldsymbol{n} \cdot \boldsymbol{u} = 0$	$no\ flux$	$no\ flux$
5	$no\ flux$	$no\ flux$	$no\ flux$	-	-	$no\ flux$
6	$no\ flux$	$no\ flux$	$no\ flux$	$-\boldsymbol{n} \cdot \boldsymbol{u} = 0$	$no\ flux$	$no\ flux$
7	$no\ flux$	-	-	$-\boldsymbol{n} \cdot \boldsymbol{u} = 0$	$no\ flux$	$no\ flux$
8	-	-	-	-	$\omega_i = \omega_{i,in}$	$T = T_{op}$
9	-	-	-	-	-	$no\ flux$
10	-	-	-	-	-	$Periodic$
11	-	-	-	-	$no\ flux$	-
12	$\phi_s = 0$	-	-	$p = -12\mu_g/W_{channel}^2(y + index \cdot 0.5L)$*	-	-
13	-	$no\ flux$	$no\ flux$	-	-	-
14	-	-	-	$-\boldsymbol{n} \cdot \boldsymbol{u} = 0$	$no\ flux$	-
15	-	-	-	$-\boldsymbol{n} \cdot \boldsymbol{u} = 0$	$no\ flux$	-
16	-	$no\ flux$	$no\ flux$	-	-	-
17	$\phi_s = V_{applied}$	-	-	$p = -12\mu_g/W_{channel}^2(y + index \cdot 0.5L)*$	-	-
18	-	-	-	-	$-\boldsymbol{n} \cdot \rho\omega_i \sum_{\boldsymbol{k}} \widetilde{D}_{ik,ef} = 0$	$no\ flux$
19	$no\ flux$	-	-	$-\boldsymbol{n} \cdot \boldsymbol{u} = 0$	$no\ flux$	$no\ flux$
20	$no\ flux$	$no\ flux$	$no\ flux$	$-\boldsymbol{n} \cdot \boldsymbol{u} = 0$	$no\ flux$	$no\ flux$
21	$no\ flux$	$no\ flux$	$no\ flux$	-	-	$no\ flux$
22	$no\ flux$	$no\ flux$	$no\ flux$	$-\boldsymbol{n} \cdot \boldsymbol{u} = 0$	$no\ flux$	$no\ flux$
23	$no\ flux$	-	-	$-\boldsymbol{n} \cdot \boldsymbol{u} = 0$	$no\ flux$	$no\ flux$
24	-	-	-	-	$-\boldsymbol{n} \cdot \rho\omega_i \sum_{\boldsymbol{k}} \widetilde{D}_{ik,ef} = 0$	$no\ flux$
25	-	-	-	-	-	$no\ flux$

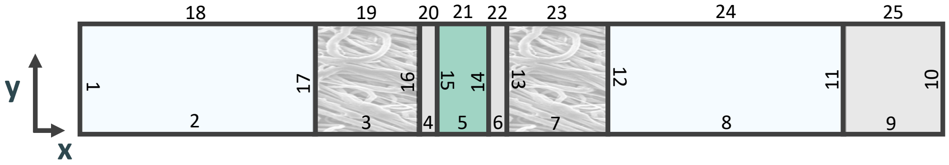

Figure S12 *Schematic overview of the boundaries for the simulation of the Full MEA configuration. The respective boundary conditions can be found in* ***Table S4****.*

*__Table S4__ Overview of boundary conditions used in simulations for the Exchange MEA configuration. A schematic overview of the corresponding boundaries is given in __Figure S13__. *index refers to the number of the sub cell in the expansion method.*

**Boundary**	**Potential distribution**	**Water transport**	**Dissolved species transport**	**Darcy's law**	**Transport of gaseous species**	**Heat transfer**
**1**	-	-	$no\ flux$	-	-	$Periodic$
**2**	-	-	$c_i = c_{i,in}$	-	-	$T = T_{op}$
**3**	$no\ flux$	-	$no\ flux$	-	-	$no\ flux$
**4**	$no\ flux$	$no\ flux$	$no\ flux$	-	-	$no\ flux$
**5**	$no\ flux$	$no\ flux$	$no\ flux$	$-\boldsymbol{n} \cdot \boldsymbol{u} = 0$	$no\ flux$	$no\ flux$
**6**	$no\ flux$	-	-	$-\boldsymbol{n} \cdot \boldsymbol{u} = 0$	$no\ flux$	$no\ flux$
**7**	-	-	-	-	$\omega_i = \omega_{i,in}$	$T = T_{op}$
**8**	-	-	-	-	-	$no\ flux$
**9**	-	-	-	-	-	$Periodic$
**10**	-	-	-	-	$no\ flux$	-
**11**	$\phi_s = 0$	-	-	$p = -12\mu_g/W_{channel}^2 (y + index \cdot 0.5L)$*	-	-
**12**	-	$no\ flux$	$no\ flux$	-	-	-
**13**	-	-	-	$-\boldsymbol{n} \cdot \boldsymbol{u} = 0$	$no\ flux$	-
**14**	$\Delta\phi_{l,Donnan} = \phi_{l,AEM} - \phi_{l,electrolyte}$	$\lambda = 17$	$\frac{c_{i,AEM}}{c_{i,electrolyte}} = exp\left(-\frac{z_i F}{RT}\Delta\phi_{l,Donnan}\right)$	-	-	-
**15**	$\phi_s = V_{applied}$	-	-	-	-	-
**16**	-	-	$-\boldsymbol{n} \cdot \nabla c_i = 0$	-	-	$no\ flux$
**17**	$no\ flux$	-	$no\ flux$	-	-	$no\ flux$
**18**	$no\ flux$	$no\ flux$	$no\ flux$	-	-	$no\ flux$
**19**	$no\ flux$	$no\ flux$	$no\ flux$	$-\boldsymbol{n} \cdot \boldsymbol{u} = 0$	$no\ flux$	$no\ flux$
**20**	$no\ flux$	-	-	$-\boldsymbol{n} \cdot \boldsymbol{u} = 0$	$no\ flux$	$no\ flux$
**21**	-	-	-	-	$-\boldsymbol{n} \cdot \rho\omega_i \sum_{\boldsymbol{k}} \widetilde{D}_{ik,eff} \boldsymbol{d}_k = 0$	$no\ flux$
**22**	-	-	-	-	-	$no\ flux$

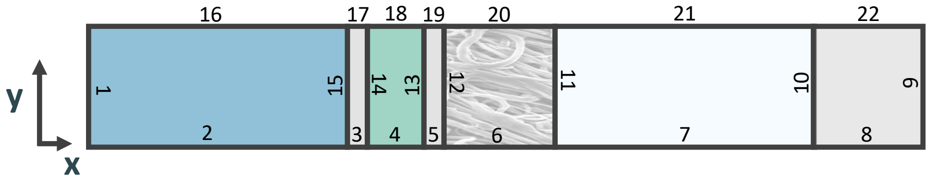

__Figure S13__ Schematic overview of the boundaries for the simulation of the Exchange MEA configuration. The respective boundary conditions can be found in __Table S5__.

7 Additional Governing Equations, Calculations for Parameters and Variables

This section provides an overview of the computed parameters and variables used in the model which are not discussed in the main body of the paper for brevity.

7.1 Governing equations gas phase

In the porous domains, fluid flow is computed using Darcy's law:

$$\boldsymbol{u}_g = -\frac{\kappa}{\mu}\nabla p \qquad \nabla\cdot\left(\rho\boldsymbol{u}_g\right) = R_m \tag{S6}$$

Here κ $[\mathrm{m}^2]$ is the permeability of the porous medium. The permeability of the diffusion medium is computed with the Tomadakis-Sotrichos equation for the permeability of randomly overlapping fibers which matches experimental work [14, 15]. The permeability in the catalytic domain follows the Carman-Kozenzy equation whereby the experimentally determined parameters of McLaughlin et al have been taken [16]. R_m $[\mathrm{kg\,m^{-3}\,s^{-1}}]$ refers to mass sources which are discussed in the section later in this section. For convenience, the viscosity of ambient air is used. The density follows from the conservation equation for gaseous species through the ideal gas law.

Within the gaseous domains, the conservation equation for the gaseous species reads:

$$\nabla\cdot(\rho\omega_i\sum_{\boldsymbol{k}}\widetilde{D}_{ik,eff}\boldsymbol{d}_k) + \rho(\boldsymbol{u}_g\cdot\nabla)\omega_i = R_i \tag{S7}$$

Here, diffusion is described with the Maxwell-Stefan model whereby ω_i is the mass fraction of species *i*, $\boldsymbol{d}_k[\mathrm{m}^{-1}]$ is the diffusional driving force acting on species *k* ($\boldsymbol{d}_k = \nabla x_k + [(x_k-\omega_k)\nabla p/p]$). $\widetilde{D}_{ik,eff}$ $[\mathrm{m^2\,s^{-1}}]$ is the binary diffusivity of species *i* and *k* corrected for porosity with a Bruggeman's relationship ($\widetilde{D}_{ik,eff} = \epsilon_p^{1.5}\widetilde{D}_{ik}$). The temperature dependent gas-phase binary diffusivities, $\widetilde{D}_{ik}$, follow from Fuller et al [17]:

$$D_{i,k} = \left(10^{-3}T\left[\frac{1}{K}\right]\right)^{1.75}\frac{\left(\frac{1}{M_{w,i}}+\frac{1}{M_{w,k}}\right)^{\frac{1}{2}}}{p\left(V_{P,i}^{0.33}+V_{P,k}^{0.33}\right)^2} \tag{S8}$$

7.2 Mass Sources

For an electrochemical reaction in the catalytic domains, the molar source term for a species *i* reads:

$$R_{i,CT} = a_{v,CL}\sum_l\frac{v_{i,l}\boldsymbol{i}_l}{n_lF} \tag{S9}$$

Wherein $a_{v,CL}$ $[\mathrm{m}^{-1}]$ is the specific surface area of the catalytic particles in the catalytic layer obtained through tomography experiments by McLaughlin et al [16]. The molar rate of consumption or production of dissolved species due to homogeneous reaction given in equations 16-20 can expressed as

$$R_{i,HR} = \sum_k v_{i,k}\left(k_k\prod_{v_{i,k}<0}c_i^{-v_{i,k}} - k_{kr}\prod_{v_{i,k}>0}c_i^{v_{i,k}}\right) \tag{S10}$$

Whereby v_{k_i} $[-]$ is the stochiometric coefficient of the reaction.

The molar rate of mass transfer of CO_2 between the gas phase and the ionomer phase in the catalytic domain is described by:

$$R_{CO_2,PT} = a_{v,CL}\frac{D_{CO_2,m}}{\delta}\left(\mathcal{H}_{CO_2}x_{CO_2}p - c_{CO_2}\right) \tag{S11}$$

Here $D_{CO_2,m}$ $[\mathrm{m^2\ s^{-1}}]$ is the diffusion coefficient of CO_2 in the ionomer phase, δ $[\mathrm{nm}]$ is the estimated diffusion length through the ionomer phase towards a catalytic particle for a particle mass fraction of 20% (See section 7.6). $\mathcal{H}_{CO_2}$ $[\mathrm{mM}]$ is Henry's temperature dependent constant and x_{CO_2} $[-]$ is the molar fraction of CO_2 in the gas phase. At the anode, dissolution of CO_2 will occur when the concentration exceeds its solubility limit governed by equation S19.

The condensation and evaporation rate of water is computed with:

$$R_{w,PT} = a_{v,CL} k_{MT,w} \left(x_w \frac{p}{p_w} - a_w \right) \tag{S12}$$

Whereby $k_{MT,w}$ $[\mathrm{mol\ m^{-2}\ s^{-1}}]$ is the mass transfer coefficient of water vapor and p_w $[\mathrm{kPa}]$ is the partial pressure of water at a certain temperature computed with the Buck equation (equation S18). An overview of the mass sources is given below in **Table S5**.

Table S5 *Overview of the considered mass sources in the simulations.*

Species	Charge transfer reactions	Homogeneous reactions	Phase transfer reactions
	Dissolved species (ionomer phase and liquid electrolyte)		
Water in ionomer	Acidic and alkaline OER, HER, COER	Equations 16, 19, 20	Equation S12
$\boldsymbol{CO_2}$	COER	Equations 16, 18	Equation S11
$\boldsymbol{H^+}$	Acidic OER	Equations 16, 17, 20	-
$\boldsymbol{OH^-}$	Alkaline OER, HER, COER	Equations 18, 19, 20	-
$\boldsymbol{HCO_3^-}$	-	Equations 16, 17, 18, 19	-
$\boldsymbol{CO_3^{2-}}$	-	Equations 17, 19	-
$\boldsymbol{K^+}$	-	-	-
	Gaseous species		
$\boldsymbol{CO_2}$	COER	-	Equation S11
$\boldsymbol{CO}$	COER	-	-
$\boldsymbol{H_2}$	HER	-	-
$\boldsymbol{H_2O}$	-	-	Equation S12

7.3 Water content in the ionomer phase

Water transport is modelled using the mathematical model developed by Weber et al [18, 19]. This framework has originally been developed for proton exchange membranes, however anion exchange membranes exhibit similar behavior [20-22]. The framework builds on the notion that two transport nodes exist, depending whether the membrane is in contact with liquid water or water vapor. The water content of the membrane is expressed with λ $[-]$, which can be calculated with:

$$\lambda = (1 - S)\lambda_v + S\lambda_L \tag{S13}$$

Here S $[-]$ is the fraction of expanded channels in the ionomer phase which is calculated with the critical channel radius r_c $[\mathrm{m}]$:

$$S = \frac{1}{2}\left[1 - \mathrm{erf}\left(\frac{\ln r_c - \ln(1.25)}{0.3\sqrt{2}}\right)\right] \tag{S14}$$

λ_L is the water content of a liquid equilibrated constant which is set at a constant value of 17. λ_v is the water content of a vapor equilibrated membrane which is a function of the simulated water activity, a_w $[-]$:

$$\lambda_v = 30.752a_w^3 - 41.194a_w^2 + 21.141a_w \tag{S15}$$

Note that the temperature dependence on the water content has been neglected since contradictory trends are reported in literature [20-23].

The water activity dependent transport coefficient $\alpha_{w,eff}$ $[\mathrm{mol^2 J^{-1} cm^{-1} s^{-1}}]$ is linearly interpolated from Peng et al, as done previously by Weng et al [22, 24]. For vapor- and liquid equilibrated transport modes respectively:

$$\alpha_{w,v} = 8 \cdot 10^{-14} exp(11.47a_w)(0.036T[K] - 9.725) \tag{S16}$$

The liquid equilibrated transport coefficient is typically significantly higher then $\alpha_{w,l}$, we adopt the method by Weng et al [24]:

$$\alpha_{w,l} = 8 \cdot 10^{-12} exp(11.47a_w)(0.036T[K] - 9.725) \tag{S17}$$

Note that membrane swelling has not been incorporated in the computational model.

7.4 Empirical temperature dependent relations

The Buck equation is used to compute the saturation vapor pressure of water:

$$p_w = 0.61121\, exp\left(\left(18.678 - \frac{T[°C]}{234.5}\right)\left(\frac{T[°C]}{257.14 + T[°C]}\right)\right)[kPa] \tag{S18}$$

The temperature dependence of Henry's constant for the solubility of CO_2 is computed:

$$\mathcal{H}_{CO_2} = 34\, exp\left(2400\left(\frac{1}{T[K]} - \frac{1}{298}\right)\right)[mM] \tag{S19}$$

The temperature dependence of the enthalpy change of water evaporation/condensation is implemented through:

$$\Delta H_{w,vap} = 2.672 \cdot 10^5 (T_{cr} - T)^{0.38}\ [J\ kg^{-1}] \tag{S20}$$

Whereby T_{cr} is the critical temperature with a value of 647.3 $[K]$ [25].

7.5 Effective properties porous media

The porosity of the diffusion media, ϵ_{DM} $[-]$, follows from Gostick et al whereby Toray 90 is chosen [15]. The permeability is computed with the Tomadakis-Sotrichos method for randomly overlapping fibres [14]:

$$k_{DM} = \frac{\epsilon_{DM}}{8\ln(\epsilon_{DM})^2} \frac{(\epsilon_{DM} - \epsilon_{DM,p})^{(\alpha_p+2)} d_f^2}{(1-\epsilon_{DM,p})^{\alpha_p}\left[(\alpha_p+1)\epsilon_{DM} - \epsilon_{DM,p}\right]^2} \tag{S21}$$

$\epsilon_{DM,p}$ $[-]$ and α_p $[-]$ are constants and d_f $[\mathrm{mm}]$ is the average fiber diameter of in the diffusion medium. Note that it is easily possible to implement the in-plane permeability since the diffusion media exhibit anisotropy. It was however found that this has effectively no effect on the modeling results since the bulk of the gaseous transport is in the through-plane direction.

The porosity of the catalytic domain (void fraction), ϵ_{CL} $[-]$ is based on the observations of McLaughlin et al [16]. It is used to compute the permeability of the domain with the Carman-Kozeny equation:

$$k_{CL} = \frac{d_p^2 \epsilon_{CL}^3}{16K_{CK}(1-\epsilon_{CL})^2} \tag{S22}$$

Where K_{CK} $[-]$ is the Carman-Kozeny constant and d_p $[\mathrm{nm}]$ is the average grain size in the CL [15].

7.6 Catalytic layer geometric properties

Based on geometric arguments and the experimental observations by Mclaughlin et al, it possible to estimate relevant properties of the catalytic domain. In particular, the ionomer fraction, solid fraction and ionomer film thickness are of interest.

Assuming the catalytic particles are spherical and are evenly coated with ionomer with a film thickness of $\delta\ [\mathrm{nm}]$, the thickness can be estimated as:

$$\delta = R_{np}\left[\left(1+\frac{\epsilon_{ionomer}}{\epsilon_{np}}\right)^{\frac{1}{3}}-1\right] \tag{S23}$$

The ratio $\frac{\epsilon_{ionomer}}{\epsilon_{np}}$ can be computed from the known mass percentage of ionomer taken from McLaughlin et al, which is 20%:

$$\frac{\epsilon_{ionomer}}{\epsilon_{np}} = \frac{Mass_{ionomer}}{Mass_{np}}\frac{\rho_{np}}{\rho_m} = 2.19$$

We find a film thickness of 10 nm. Since we know the experimental value for the porosity of the CL, ϵ_{CL}, and with the definition:

$$1 = \epsilon_{CL} + \epsilon_{ionomer} + \epsilon_{np}$$

We can easily calculate all the volume fractions. The numeric values for the computed properties are given in **Table S8**.

7.7 Diffusion coefficients in water

The temperature dependent diffusion coefficients in water are given below in table **S6** [1].

Table S6 *Overview of the temperature dependent diffusion coefficients of dissolved species*

Aqueous species *i*	Diffusion coefficient, $D_{i,w}$ $[m^2s^{-1}]$
H^+	$4.49\cdot 10^{-9} exp\left(-1430\left(\frac{1}{T[K]}-\frac{1}{273.15}\right)\right)$
OH^-	$2.89\cdot 10^{-9} exp\left(-1750\left(\frac{1}{T[K]}-\frac{1}{273.15}\right)\right)$
K^+	$1.957\cdot 10^{-9} exp\left(-2300\left(\frac{1}{T[K]}-\frac{1}{298.15}\right)\right)$
HCO_3^-	$7.016\cdot 10^{-9}\left(\frac{T[K]}{204.0282}-1\right)^{2.3942}$
CO_3^2-	$5.447\cdot 10^{-9}\left(\frac{T[K]}{210.2646}-1\right)^{2.1929}$
CO_2	$2.17\cdot 10^{-9} exp\left(-2345\left(\frac{1}{T[K]}-\frac{1}{303}\right)\right)$

7.8 Diffusion in the ionomer phase – Adjusting for water content

The effective diffusion coefficients for the dissolved species in the ionomer phase are computed following the method implemented by Weng et al which hare based on the work by Grew et al [1, 26, 27]. The correction, given in equation 15, requires the computation of several membrane water content dependent variables, namely: $\varepsilon_L\ [-]$ is the volume fraction of water, $x_w\ [-]$ is the water mole fraction and $\zeta_i\ [-]$ is the ratio of species-water and species-membrane interaction. These variables are computed with:

$$\varepsilon_w = \frac{\lambda}{1+\lambda} \quad \text{(S24)}$$

$$x_w = \frac{\lambda V_m}{\lambda V_m + V_m} \quad \text{(S25)}$$

$$\zeta_i = \frac{1}{\lambda}\left(\frac{V_m}{V_w}\right)^{\frac{2}{3}}\left(\frac{M_{i,m}}{M_{i,w}}\right)^{\frac{1}{2}} \quad \text{(S26)}$$

Here $V_m = 1/(IEC \cdot \rho_m)$ $[\mathrm{m^3\ mol^{-1}}]$, which is the molar volume of the membrane, $M_{i,m}$ $[\mathrm{kg\ mol^{-1}}]$ is the reduced molar mass computed with $M_{i,m} = (1/M_i + 1/M_m)^{-1}$ and V_w $[\mathrm{m^3\ mol^{-1}}]$ is the is the molar volume of water.

7.9 Effective thermal properties

For the anolyte compartment, the thermal properties of liquid water are used whereby the built-in material library is used which accounts for a temperature dependency (COMSOL Multiphysics v5.6). The heat capacity of $KHCO_3$-water solutions lacks published data, especially regarding temperature dependence. However, the heat capacity of water is generally higher than the heat capacity of an electrolyte, which means that the thermal capacity of water taken here is, if differing significantly at all, a conservative minimum approximation of the temperature field. Also, the viscosity of the anolyte is assumed to be the viscosity of pure water as the differences remain of secondary relevance. Closer modeling of the viscosity of $KHCO_3$-containing solution can be found in Pereira et al. [28]. The gas fraction of any bubbles that may built up in the anolyte is neglected, as is the influence of bubble induced convection. For the gas phase, which occupies the gas channels, the diffusion media and the catalytic layers, the thermal properties of air are used. Here, the values are adjusted for the water vapor fraction since the thermal properties of water vapor are significantly different and the water mass fraction is expected to vary significantly spatially. It is possible to approximate the thermal conductivity, density and heat capacity for gaseous mixtures, however due to computational constraints this was neglected in this work [29]. The adjustment for water vapor fraction is easily implemented through COMSOL's *moist air* option within the *Heat Transfer in Porous Media* module.

The thermal properties of multiphase domains (diffusion media, catalytic layers, ionomer phase) are volume averaged. The properties of the individual constituents can be found in **Table S8**. The thermal conductivity of the membrane is volume averaged with the computed volume fraction of water in the membrane, x_w $[-]$ [30] :

$$\kappa_m = x_w \kappa_w + (1 - x_w)\kappa_{m,dry}$$

Whereby κ_w $[\mathrm{W\ m^{-1}\ K^{-1}}]$ is the conductivity of liquid water and $\kappa_{m,dry}$ $[\mathrm{W\ m^{-1}\ K^{-1}}]$ is the thermal conductivity of dehydration Nafion.

7.10 Homogeneous Reactions Rate Extrapolations

The temperature dependence of the homogeneous reactions in the electrolyte- and ionomer domains has been implemented through inter- and extrapolations from literature. The temperature dependent equilibration constant for water dissociation is taken from Sweeton et al [31] whereby the correction for water activity has been neglected:

$$\log(K_w) = \frac{3.46917 \cdot 10^4}{T} + 105.151\ln(T) - 0.1075733\,T - \frac{2.35812 \cdot 10^6}{T^2} - 6.70857 \cdot 10^2 \quad \text{(S27)}$$

The equilibrium constant for the homogeneous reactions (equations 16-20) are taken from Roy et al [32]:

$$\ln(K_1) = 2.83655 - \frac{2307.1266}{T} - 1.5529413 \ln(T) \tag{S28}$$

$$\ln(K_2) = -9.226508 - \frac{3351.6106}{T} - 0.2005743 \ln(T) \tag{S29}$$

The temperature dependence of the rate constants are computed through interpolations from Schulz et al [3]. These have been fitted with experimental data up to 35 ºC with the follow in expressions.

$$\mathrm{k}_1 = \exp(1246.98 - 6.19 \cdot 10^4/T - 183\ln(T)) \tag{S30}$$

$$k_2 = 499002.24 \exp(-90166.83/RT)/K_w \tag{S31}$$

The temperature dependence for k_1 and k_3 are extrapolated above this temperature range with 2nd order polynomials. An overview of the used rate expressions is given in **Table S7**.

Table S7 *Temperature dependence of the rate constants for the homogeneous reactions occurring in the liquid- and ionomer domains. The expressions have been adopted from Schulz et al [3]. *Extrapolated from original expression above 35 ºC.*

Rate constant	Calculation	Units
$\boldsymbol{k_1}$*	$0.0000472\,T^2 - 0.0249478\,T + 3.2836$	$[s^{-1}]$
$\boldsymbol{k_{1r}}$	k_1/K_1	$[m^3\,mol^{-1}\,s^{-1}]$
$\boldsymbol{k_2}$	$k_{2r}K_2$	$[s^{-1}]$
$\boldsymbol{k_{2r}}$	$5 \cdot 10^{10}$	$[l\,mol^{-1}\,s^{-1}]$
$\boldsymbol{k_3}$*	$10.28\,T^2 - 5864.60\,T + 812921$	$[l\,mol^{-1}\,s^{-1}]$
$\boldsymbol{k_{3r}}$	k_3K_w/K_1	$[s^{-1}]$
$\boldsymbol{k_4}$	$6 \cdot 10^9$	$[l\,mol^{-1}\,s^{-1}]$
$\boldsymbol{k_{4r}}$	k_4K_w/K_2	$[s^{-1}]$
$\boldsymbol{k_w}$	$1.4 \cdot 10^{-3}$	$[mol\,l^{-1}\,s^{-1}]$
$\boldsymbol{k_{wr}}$	k_w/K_w	$[m^3\,mol^{-1}\,s^{-1}]$

8 Simulation Parameters

Table S8 *Overview of all the relevant modeling parameters used in this work.*

Parameter	Description	Value	Unit	Ref.
	Dimensions			
$W_{channel}$	Flow channel width	0.5	mm	-
W_{DM}	Diffusion medium width	0.2	mm	-
W_{CL}	Catalyst layer width	0.005	mm	-
W_{M}	Membrane width	0.05	mm	-
W_{BP}	Bipolar plate width	5	mm	-
L_{cell}	Length sub cell expansion method	0.1	mm	-
$W_{total,Full\,MEA}$	Total domain length Full MEA model	1.96	mm	-
$W_{total,Exchange\,MEA}$	Total domain length Exchange MEA model	1.76	mm	-
	Catalyst layer properties			
ϵ_{CL}	Porosity (void volume fraction) catalyst layer	0.68	-	[16]
$\epsilon_{ionomer}$	Ionomer volume fraction catalyst layer	0.22	-	-
ϵ_{np}	Nanoparticle volume fraction catalyst layer	0.1	-	-
R_{np}	Radius catalytic nanoparticles	30	nm	[16]
δ	Ionomer film thickness catalyst layer	10	nm	-
ρ_{np}	Density catalytic nanoparticles	10.5	$g\,cm^{-3}$	[33]
k_{CL}	Permeability CL, computed with equation S22	$4.42\cdot 10^{-16}$	m^2	[15]
d_p	Average grainsize CL	124	nm	[15]
$a_{v,CL}$	Specific surface area catalyst layer	6E6	m^{-1}	[16]
$\sigma_{s,CL}$	Electrical conductivity catalyst layer	100	$S\,m^{-1}$	[34]
$k_{MT,w}$	Mass transfer coefficient water vapor	0.06	$mol\,m^{-2}s^{-1}$	[24]
κ_{np}	Thermal conductivity catalyst layer particles	0.2	$W\,m^{-1}\,K^{-1}$	[35]
$C_{p,CL}$	Heat capacity catalyst layer	2000	$J\,kg^{-1}\,K^{-1}$	[1]
	Diffusion medium properties			
ϵ_{DM}	Porosity diffusion medium	0.8	-	*[15]*
d_f	Fiber diameter diffusion medium	0.005	mm	*[15]*
k_{DM}	Permeability diffusion medium	$1.1268\cdot 10^{-11}$	m^2	-
$\sigma_{s,DM}$	Electrical conductivity diffusion medium	1250	$S\,m^{-1}$	*[36]*
κ_{DM}	Thermal conductivity diffusion medium	0.2	$W\,m^{-1}\,K^{-1}$	*[25]*
$C_{p,DM}$	Heat capacity diffusion medium	1000	$J\,kg^{-1}\,K^{-1}$	*[1]*
ρ_{DM}	Density diffusion medium	300	$kg\,m^{-3}$	*[1]*
	Membrane/ionomer phase properties			
IEC	Ion exchange capacity	1.7	$mmol\,g^{-1}$	[1]
q_{fix}	Fixed back charge AEM	$3.2805\cdot 10^{8}$	$C\,m^{-3}$	[1]
ξ_v	Effective electro-osmotic coefficient, vapor equilibrated	0.61	-	[24]
ξ_l	Effective electro-osmotic coefficient, liquid equilibrated	3	-	[24]
ρ_m	Hydrated membrane density	2	$g\,ml^{-1}$	[23]
$C_{p,CL}$	Heat capacity membrane	4000	$J\,kg^{-1}\,K^{-1}$	[1]
$\kappa_{m,dry}$	Thermal conductivity dry membrane	0.16	$W\,m^{-1}\,K^{-1}$	[30]
	Kinetics			
U^0_{HER}	Standard reduction potential HER	0	V	[1]
U^0_{COER}	Standard reduction potential COER	-0.11	V	[1]
U^0_{OER}	Standard reduction potential OER	1.23	V	[1]
A_{HER}	Pre-exponent factor exchange current density, HER	$8.84\cdot 10^{6}$	$mA\,cm^{-2}$	[1]
A_{COER}	Pre-exponent factor exchange current density, COER	$7.25\cdot 10^{8}$	$mA\,cm^{-2}$	[1]
$A_{OER,acidic}$	Pre-exponent factor exchange current density, acidic OER	$9.40\cdot 10^{-7}$	$mA\,cm^{-2}$	[1]
$A_{OER,alkaline}$	Pre-exponent factor exchange current density, alkaline OER	$1.23\cdot 10^{-4}$	$mA\,cm^{-2}$	[1]
$E_{a,HER}$	Activation energy exchange current density, HER	$(83+pH)$	$kJ\,mol^{-1}$	[1]
$E_{a,COER}$	Activation energy exchange current density, COER	100	$kJ\,mol^{-1}$	[1]
$E_{a,OER}$	Activation energy exchange current density, OER	$(11+pH)$	$kJ\,mol^{-1}$	[1]
$\alpha_{c,HER}$	Cathodic charge transfer coefficient, HER	0.44	-	[1]
$\alpha_{c,COER}$	Cathodic charge transfer coefficient, COER	1	-	[1]
$\alpha_{a,OER}$	Anodic charge transfer coefficient, OER	1.5	-	[1]

$C_O^{\gamma_{HER}}$	Concentration dependence, HER	$-$	-	[1]
$C_O^{\gamma_{COER}}$	Concentration dependence, COER	$(c_{CO2}/1[M])^{1.5}$	-	[1]
$C_R^{\gamma_{OER,acidic}}$	Concentration dependence, acidic OER	$-$	-	[1]
$C_R^{\gamma_{OER,alkaline}}$	Concentration dependence, alkaline OER	$(c_{OH-}/1[M])$	-	[1]
		Initial concentrations dissolved species membrane - Full MEA		
$c_{H+,m,0}$	Equilibrated with 0.5 M $KHCO_3$ at ambient conditions	$1.132 \cdot 10^{-5}$	mM	-
$c_{OH-,m,0}$	Equilibrated with 0.5 M $KHCO_3$ at ambient conditions	$6.085 \cdot 10^{-4}$	mM	-
$c_{K+,m,0}$	Equilibrated with 0.5 M $KHCO_3$ at ambient conditions	75.571	mM	-
$c_{HCO_3-,m,0}$	Equilibrated with 0.5 M $KHCO_3$ at ambient conditions	3278.089	mM	-
$c_{CO_32-,m,0}$	Equilibrated with 0.5 M $KHCO_3$ at ambient conditions	98.761	mM	-
$c_{CO_2,m,0}$	Equilibrated with 0.5 M $KHCO_3$ at ambient conditions	38.399	mM	-
		Enthalpy changes reactions		
ΔH_1	Enthalpy change homogeneous reaction 1	9.160	$\mathrm{kJ\ mol^{-1}}$	-
ΔH_2	Enthalpy change homogeneous reaction 2	14.700	$\mathrm{kJ\ mol^{-1}}$	-
ΔH_3	Enthalpy change homogeneous reaction 3	-46.655	$\mathrm{kJ\ mol^{-1}}$	-
ΔH_4	Enthalpy change homogeneous reaction 4	-41.114	$\mathrm{kJ\ mol^{-1}}$	-
ΔH_w	Enthalpy change water dissociation	55.815	$\mathrm{kJ\ mol^{-1}}$	-
		Peltier coefficients		
Π_{HER}	Peltier coefficient HER	$13\,T[K]/298$	mV	[1]
Π_{COER}	Peltier coefficient COER	$40\,T[K]/298$	mV	[1]
Π_{OER}	Peltier coefficient OER	$240\,T[K]/298$	mV	[1]
		Bipolar plate properties		
κ_{BP}	Thermal conductivity aluminum	238	$\mathrm{W\ m^{-1}K^{-1}}$	[37]
ρ_{BP}	Density aluminum	2700	$\mathrm{kg\ m^{-3}}$	[37]
$C_{p,BP}$	Heat capacity aluminum	900	$\mathrm{J\ kg^{-1}K^{-1}}$	[37]

9 Bibliography

1. Weng, L.-C., A.T. Bell, and A.Z. Weber, *Towards membrane-electrode assembly systems for CO2 reduction: a modeling study.* Energy & Environmental Science, 2019. **12**(6): p. 1950-1968.
2. Hansen, K.U., L.H. Cherniack, and F. Jiao, *Voltage Loss Diagnosis in CO2 Electrolyzers Using Five-Electrode Technique.* ACS Energy Letters, 2022. **7**: p. 4504-4511.
3. Schulz, K.G., et al., *Determination of the rate constants for the carbon dioxide to bicarbonate inter-conversion in pH-buffered seawater systems.* Marine Chemistry, 2006. **100**(1-2): p. 53-65.
4. Yin, Z.L., et al., *An alkaline polymer electrolyte CO electrolyzer operated with pure water.* Energy & Environmental Science, 2019. **12**(8).
5. Crandall, B.S., et al., *Kilowatt-scale tandem CO2 electrolysis for enhanced acetate and ethylene production.* Nature Chemical Engineering, 2024. **1**(6): p. 421-429.
6. Endrodi, B., et al., *High carbonate ion conductance of a robust PiperION membrane allows industrial current density and conversion in a zero-gap carbon dioxide electrolyzer cell.* Energy & Environmental Science, 2020. **13**(11): p. 4098-4105.
7. Endrödi, B., et al., *Operando cathode activation with alkali metal cations for high current density operation of water-fed zero-gap carbon dioxide electrolysers.* Nature Energy, 2021. **6**(4): p. 439-448.
8. Li, W.Z., et al., *Bifunctional ionomers for efficient co-electrolysis of CO and pure water towards ethylene production at industrial-scale current densities.* Nature Energy, 2022. **7**(9): p. 835-843.
9. Blake, J.W., et al., *Inhomogeneities in the Catholyte Channel Limit the Upscaling of CO2 Flow Electrolysers.* Acs Sustainable Chemistry & Engineering, 2023. **11**(7): p. 2840-2852.
10. Pant, L.M., et al., *Along-the-channel modeling and analysis of PEFCs at low stoichiometry: Development of a 1+ 2D model.* Electrochimica Acta, 2019. **326**: p. 134963.
11. *The Tertiary Current Distribution, Nernst-Planck Interface*. [cited 2023 24-11-2023]; Available from: https://doc.comsol.com/5.6/doc/com.comsol.help.battery/battery_ug_electrochem.07.018.html .
12. Sastre, M. and J.A. Santaballa, *A Note on the Meaning of the Electroneutrality Condition for Solutions.* Journal of Chemical Education, 1989. **66**(5): p. 403-404.
13. Kodym, R., D. Snita, and K. Bouzek, *Mathematical Modeling of Electromembrane Processes.* Current Trends and Future Developments on (Bio-) Membranes: Membrane Desalination Systems: The Next Generation, 2019: p. 285-326.
14. Tomadakis, M.M. and T.J. Robertson, *Viscous permeability of random fiber structures: Comparison of electrical and diffusional estimates with experimental and analytical results.* Journal of Composite Materials, 2005. **39**(2): p. 163-188.
15. Gostick, J.T., et al., *In-plane and through-plane gas permeability of carbon fiber electrode backing layers.* Journal of Power Sources, 2006. **162**(1): p. 228-238.
16. McLaughlin, D., et al., *Tomographic Reconstruction and Analysis of a Silver CO2 Reduction Cathode.* Advanced Energy Materials, 2020. **10**(19).
17. Fuller, E.N., P.D. Schettle, and J.C. Giddings, *A New Method for Prediction of Binary Gas-Phase Diffusion Coeffecients.* Industrial and Engineering Chemistry, 1966. **58**(5): p. 19-+.
18. Weber, A.Z. and J. Newman, *Modeling transport in polymer-electrolyte fuel cells.* Chemical Reviews, 2004. **104**(10): p. 4679-4726.
19. Weber, A.Z. and J. Newman, *Transport in polymer-electrolyte membranes - II. Mathematical model.* Journal of the Electrochemical Society, 2004. **151**(2): p. A311-A325.

20. Duan, Q.J., S.H. Ge, and C.Y. Wang, *Water uptake, ionic conductivity and swelling properties of anion-exchange membrane.* Journal of Power Sources, 2013. **243**: p. 773-778.
21. Li, Y.S., T.S. Zhao, and W.W. Yang, *Measurements of water uptake and transport properties in anion-exchange membranes.* International Journal of Hydrogen Energy, 2010. **35**(11): p. 5656-5665.
22. Peng, J., et al., *Effect of CO absorption on ion and water mobility in an anion exchange membrane.* Journal of Power Sources, 2018. **380**: p. 64-75.
23. Kusoglu, A. and A.Z. Weber, *New Insights into Perfluorinated Sulfonic-Acid Ionomers.* Chemical Reviews, 2017. **117**(3): p. 987-1104.
24. Weng, L.C., A.T. Bell, and A.Z. Weber, *A systematic analysis of Cu-based membrane-electrode assemblies for CO(2)reduction through multiphysics simulation.* Energy & Environmental Science, 2020. **13**(10): p. 3592-3606.
25. Birgersson, E., M. Noponen, and M. Vynnycky, *Analysis of a two-phase non-isothermal model for a PEFC.* Journal of the Electrochemical Society, 2005. **152**(5): p. A1021-A1034.
26. Grew, K.N. and W.K.S. Chiu, *A Dusty Fluid Model for Predicting Hydroxyl Anion Conductivity in Alkaline Anion Exchange Membranes.* Journal of the Electrochemical Society, 2010. **157**(3): p. B327-B337.
27. Grew, K.N., X.M. Ren, and D. Chu, *Effects of Temperature and Carbon Dioxide on Anion Exchange Membrane Conductivity.* Electrochemical and Solid State Letters, 2011. **14**(12): p. B127-B131.
28. Pereira, G., et al., *Kinematic viscosity prediction for aqueous solutions with various solutes.* Chemical Engineering Journal, 2001. **81**(1-3): p. 35-40.
29. Mason, E.A. and S.C. Saxena, *Approximate Formula for the Thermal Conductivity of Gas Mixtures.* Physics of Fluids, 1958. **1**(5): p. 361-369.
30. Khandelwal, M. and M.M. Mench, *Direct measurement of through-plane thermal conductivity and contact resistance in fuel cell materials.* Journal of Power Sources, 2006. **161**(2): p. 1106-1115.
31. Sweeton, F.H., R.E. Mesmer, and C.F. Baes, *Acidity Measurements at Elevated Temperatures. VII. Dissociation of Water.* Journal of Solution Chemistry, 1974. **3**(3): p. 191-214.
32. Roy, R.N., et al., *The Dissociation-Constants of Carbonic-Acid in Seawater at Salinities 5 to 45 and Temperatures 0-Degrees-C to 45-Degrees-C.* Marine Chemistry, 1993. **44**(2-4): p. 249-267.
33. *Silver Nanoparticles*. Available from: https://www.americanelements.com/silver-nanoparticles-7440-22-4.
34. Du, C.Y., et al., *Effective protonic and electronic conductivity of the catalyst layers in proton exchange membrane fuel cells.* Electrochemistry Communications, 2004. **6**(5): p. 435-440.
35. Ahadi, M., et al., *Thermal conductivity of catalyst layer of polymer electrolyte membrane fuel cells: Part 1-Experimental study.* Journal of Power Sources, 2017. **354**: p. 207-214.
36. *Toray Carbon Paper 090*. Available from: https://www.fuelcellstore.com/toray-carbon-paper-090.
37. Lide, D.R., *CRC handbook of chemistry and physics*. Vol. 85. 2004: CRC press.
